# T Cell Vaccination Benefits Relapsing Progressive Multiple Sclerosis Patients: A Randomized, Double-Blind Clinical Trial

**DOI:** 10.1371/journal.pone.0050478

**Published:** 2012-12-14

**Authors:** Dimitrios Karussis, Hagai Shor, Julia Yachnin, Naama Lanxner, Merav Amiel, Keren Baruch, Yael Keren-Zur, Ofra Haviv, Massimo Filippi, Panayiota Petrou, Shalom Hajag, Urania Vourka-Karussis, Adi Vaknin-Dembinsky, Salim Khoury, Oded Abramsky, Henri Atlan, Irun R. Cohen, Rivka Abulafia-Lapid

**Affiliations:** 1 Department of Neurology, MS Center and the Agnes-Ginges Center for Neurogenetics, Hadassah-Hebrew University Hospital, Jerusalem, Israel; 2 Department of Biophysics and Nuclear Medicine, Human Biology Research Center, Hadassah-Hebrew University Hospital, Jerusalem, Israel; 3 San Raffaelle Hospital, MS Center, Milan, Italy; 4 Department of Immunology, The Weizmann Institute of Science, Rehovot, Israel; Escola Paulista de Medicina - UNIFESP, Brazil

## Abstract

**Background:**

T-cell vaccination (TCV) for multiple sclerosis (MS) refers to treatment with autologous anti-myelin T-cells, attenuated by irradiation. Previously published clinical trials have been all open-labeled.

**Aim:**

To evaluate the safety and efficacy of TCV in progressive MS, in a double-blind, controlled clinical trial.

**Methodology:**

Twenty-six patients with relapsing-progressive MS were enrolled in the study (mean age: 39±9.8 years; mean EDSS: 4.4±1.7). T-cell lines reactive to 9 different peptides of the myelin antigens, MBP, MOG and PLP were raised from the patients' peripheral blood. The patients were randomized into two groups: 19 were treated with TCV (four subcutaneous injections of 10–30×10^6^ T-cells, attenuated by irradiation, on days 1, 30, 90 and 180) and 7 patients were treated with sham injections. Twenty-four patients (17 in the TCV group and 7 in the placebo) were eligible for per-protocol analysis.

**Results:**

At one year following the inclusion, an increase in the EDSS (+0.50) and an increase in 10-meter walking time (+0.18 sec), were observed in the placebo group; in the TCV group there was a decrease in the EDSS (−0.44; p<0.01) and in the 10-meter walking time (0.84 sec; p<0.005). Sixteen of the 17 patients (94.1%) in the TCV group remained relapse-free during the year of the study, as compared to 42.9% in the placebo group (p = 0.01 and p = 0.03 with adjustment). The proportion of patients with any relapse during the year of the study in the TCV-group, was reduced by 89.6%., as compared to the placebo-treated group. MRI parameters did not change significantly.

**Conclusions:**

This is the first controlled, double-blind trial with TCV in progressive MS. The results demonstrate the feasibility and safety of the procedure, and provide significant indications of clinical efficacy. Further studies with larger groups of subjects are warranted.

**Trial Registration:**

ClinicalTrials.gov NCT01448252

## Introduction

Multiple sclerosis (MS) is a chronic disease of the central nervous system (CNS) characterized by progressive loss of motor and sensory nerve function and immune-mediated inflammation and demyelization [1].

It is believed that autoimmune Th1 T-cells specific for encephalitogenic myelin antigens, including myelin basic protein (MBP), proteolipid protein (PLP) and myelin oligodendrocyte glycoprotein (MOG) play a major role in the pathogenesis of MS, irrespective of the initial triggering event [2]. These findings provide the rationale for using T-cell vaccination (TCV) to induce down-regulation of the pathogenic T-cells in MS.

TCV was first introduced in 1981 in the EAE rat model of MS using activated and irradiated MBP-specific T-cell lines and clones [3]. TCV was found to suppress EAE and induced the generation of regulatory T-cells (CD4 or CD8) specific for the anti-myelin T-cell receptors of the T-cells that were used in the vaccine, a phenomenon described as anti-idiotypic regulation [4]. Regulatory T-cells were also found to respond to molecules expressed by activated T cells, acting thus as anti-ergotypic regulators [5]. Such T-cell ergotopes included CD25 [6], HSP60 and the TNFa receptor epitopes [7]. Subsequently, regulatory networks involving CD4 and CD8 T-cells were demonstrated in rats following TCV [8]. These T-cell interactions showed MHC restriction to standard Class II and Class I antigens and to non-standard Q1a antigens [9].

Seven clinical trials of TCV at various stages of MS have been reported, and six additional trials are in progress or have recently been completed, but not yet reported (reviewed by Hellings, Raus and Stinissen [10] and by Vandenbark and Abulafia-Lapid [11]). However, none of these studies have been classified as double-blinded and placebo controlled.

Our study is a double-blind, sham-controlled trial to evaluate the safety and efficacy of TCV in progressive MS patients. We carried out multiple autologous T-cell vaccinations with a mixture of attenuated T-cell lines reactive to three or more, among 9 different peptides from the sequences of the three major myelin proteins implicated as autoantigens in MS: MBP, PLP and MOG. The trial subjects suffered from relapsing-progressive MS with progression of at least one degree in the EDSS scale during the year before the study. T-cell lines were prepared from all of the subjects, but the TCV treatments were given only in the active treatment group following randomization; the patients in the sham/placebo received saline injections instead of the cell lines. TCV-treated subjects received 10–30 million attenuated T-cells by subcutaneous injections on days 0, 30, 90, 180, and the clinical effects were measured at one year.

## Methods

The protocol for this trial and supporting CONSORT checklist are available as supporting information; see Checklist S1 and Protocol S1.

### 1. Participants

Thirty patients with definite MS and relapsing-progressive course were initially enrolled in the study. All patients signed an informed consent approved by the Institutional Review Board and the National Ministry of Health Review Board of Israel. All patients had severe progression in their functional status (increase of at least one degree in the EDSS scale) during the year prior to inclusion. The included patients were tested for their peripheral blood lymphocyte reactivites against 9 different peptides of MBP, PLP and MOG (the major known autoantigens in MS pathogenesis). T-cell lines specific for the peptides with the highest reactivity in each individual, were prepared from all the patients. The calculation of the sample size was based on the following assumption: If the relapse rate in the placebo group would be 0.8/year (as was the mean of the preselected 54 patients from our database), the power of the study would be 88% to detect a 75% reduction of the relapse rate in the TCV group with an 1∶1 design (ie 15 patients in each group, as initially intended). For a similar anticipated efficacy, the power would be 85% for a 1∶2 (placebo vs TCV) design. As mentioned, the initial design was 1∶1 (placebo∶TCV) but the Ethics committee of our Institution requested to have more patients (1∶2) assigned to the active treatment group. Such change in the design reduced somewhat the power of the study, but the total number of the patients could not increase, due to the limitations of the license from the Israeli Ministry of Health.

All randomized patients were not aware of their group allocation (including the four who finally did not enter the study).

For the initial, pre-randomization process, we scanned our patients database at the Hadassah MS Center and calculated several disease parameters using the Excel and the SPSS15 statistical software, to identify a rather homogeneous group of candidate patients according to various disease characteristics (EDSS, progressive disease with relapses, age range and relapse rates). Thereafter, the patients from the previously formulated group (54 patients) were asked if they would be interested to participate in the trial. Thirty of them who were finally selected and confirmed their willingness to participate (signing and informed consent), were randomized into the two groups (2∶1 treatment vs placebo). The final randomization was done with the help of an external monitoring agent (Dr Moshe Neuman, B.R.D, Bio-Medical Research Design: www.brd.co.il) based on only 2 parameters, EDSS and age.

Additional secondary disease characteristics which were registered but not used as the primary stratification parameters included the annual relapse rate and the duration of the disease.

**Figure 1:**
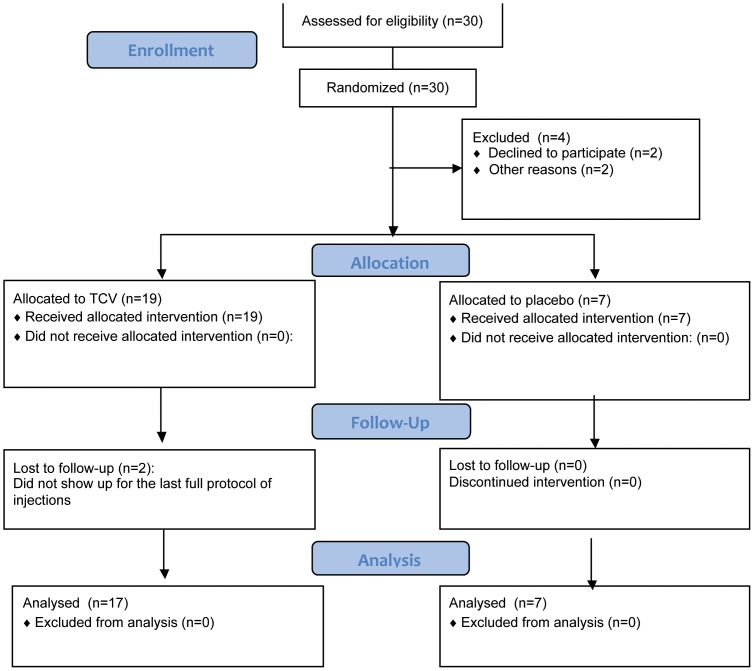
Consort flow chart.

Twenty six of them (n = 26, 15 males, 11 females; mean age of 39.07±9.81) with a mean EDSS at inclusion of 4.42±1.66 (range: 3.0–7.0) and disease duration of 8.61±4.63 years, were finally included in the study. Four patients out of initial number of 30 patients (3 assigned to placebo and one in TCV) were not included due to withdrawal of their consent or lost to follow up, before the day 1 of the trial. Of these 26 patients, 19 were assigned to the TCV-treatment group and 7 to the sham treatment/placebo group (see Figure 1: Flowchart of the trial).

Patients from the first (TCV) group were treated on days 0, 30, 90, 180 with subcutaneous TCV injections, composed of 10–30 million attenuated T-cells; patients from the second (placebo) group, received at the same time points saline injections instead of the cell lines.

Our treatment protocol included multiple autologous T-cell vaccinations with a mixture of attenuated T-cell lines reactive to three or more peptides, those which showed the highest proliferation rates in each individual patient (patient-tailored vaccine). The whole study design was double-blinded. All patients were followed up clinically and neuroradiologically for a period of one year and examined at 1, 3, 6, 9 and 12 months visits by two neurologists, the treating physician who recorded adverse events and the evaluating physician who performed the neurological examination, the EDSS status recording and the additional functional tests, which included, the 10-meter walking test, the 9-hole peg test and the PASAT cognitive test. Both neurologists were “blinded” concerning the treatment arm to which each patient was assigned. MRI was performed before the TCV injection and at 12 months. The duration of the study was one year. Two patients from the 19 in the TCV group withdrew from the study (lost to follow up). These patients did not receive the full protocol of TCV injections. Since an ITT analysis including patients who either did not show at all on visit 1, or did not receive the full treatment protocol, would be less meaningful in such a small study, all the analyses between the groups were performed for the 24 (17 in TCV and 7 in the placebo arms) patients who received the treatment protocol (per protocol analysis). The baseline demographic data of these 24 patients, are shown in Table 1. As can be seen in the Table, the two groups did not differ significantly to any of the baseline parameters (age, EDSS, relapse rate).

**Table 1:** Demographic data of the patients at inclusion to the study (N = 24).

	TCV (n = 17)	Placebo (n = 7)	Statistical test and significance
Age (mean±SD)	40.3±10.1	39.6±7.9	Mann-Whitney test, p = 0.95
Gender	8M/9F	5M/2F	Fisher's Exact test, p = 0.39
Disease Duration (years)	8.6±4.3	10.6±4.3	Mann-Whitney test, p = 0.35
EDSS	4.38±1.54	4.86±2.14	Mann-Whitney test, p = 0.49
Relapses per patient during the year preceding the inclusion	0.71±0.75	0.82±0.72	Mann-Whitney test, p = 0.76

### 2. Study Design

#### a. Inclusion Criteria

1. Clinically definite MS (according to Poser's criteria) of the relapsing-progressive type (RPMS).
2. Age: 18–60.
3. EDSS: 3.0 to 7.0.
4. Disease duration: >1 year.
5. Evidence of disease progression of 1 degree in the EDSS scale, or at least two severe relapses (requiring hospitalization and treatment) during the year prior to inclusion.
6. MRI of the brain with at least 5 lesions in the white matter (T2-weighted imaging).
7. Failure to benefit from other existing treatments according to the guidelines of the Israeli Ministry of Health.

#### b. Exclusion Criteria

1. Patients with other systemic active disease.
2. Patients who had been treated with immunosuppressive drugs during the 3–6 months depending on the cytotoxicity of the medication used prior to the inclusion.
3. Patients who previously received cellular immunotherapy or participated in other experimental protocols.
4. Pregnant women or women who do not use efficacious contraception (oral contraception, or intra-uterine device).
5. Patients with an additional autoimmune condition unrelated to MS or significant allergy.
6. Patients who cannot fully understand the treatment protocol or are unable to sign the informed consent, or in whom the clinician believes that a follow-up period of at least 12 months will not be possible.

#### c. Study Certification

The protocol was submitted and approved by the Institutional Review Board at Hadassah Hebrew University Hospital and by the National Israeli Ministry of Health Review Board of Jerusalem, Israel. The protocol included the T-cell preparation procedure, the TCV-protocol manual, the informed consent (IC), forms and the investigator's brochure (IB), Standard Operation Procedures (SOP) and Batch records (BR) filed for each patient. Clinical Case Report Forms (CRF) were prepared and filed by two neurologists for each patient's visits (total of 14 visits). Cell cultures and laboratory work were performed according to the FDA clean room regulations. All lab workers were trained and certified for clean room work. A Certified Record Controller (CRA) monitored the trial.

#### d. Clinical Efficacy Parameters

(all tested at one year –end of trial- and compared to the baseline values):

1. Change in the disability status by the EDSS disability scale, the timed 10-meters walking, the 9-hole peg test for hands dexterity and the Paced auditory serial addition test (PASAT) for cognitive impairment.
2. Change in the relapse rate and the percentages of patients with 0, 1, 2 or more relapses, during the one year follow up in the study, compared to the year prior to entry into the study.
3. Changes in various MRI parameters: i. the total burden (volume) of hyperintense lesions in T2-weighted imaging, ii. the number and total volume gadolinium-enhancing lesions in T1-weigthed imaging and, iii. the degree of cortical atrophy and axonal loss (as evidenced by the measurement of the total brain parenchymal volume).
4. Effects on the immune responses: the absolute numbers and proportions of anti-myelin reactive lymphocytes in the peripheral blood were tested pre- and post TCV.

The duration of the study was one year from the first injection.

### 3. Screening for reactivity against myelin antigens

Thirty patients with relapsing-progressive MS were pre-screened and tested for their peripheral blood lymphocyte reactivities against 9 different myelin protein peptide epitopes: the MBP peptides: M1 (84–102) and M2 (143–168); the MOG peptides: MO1 (1–22), MO2 (34–56), MO3 (64–86) and MO4 (74–96); and the PLP peptides: P1 (41–58), P2 (184–199), and P3 (190–209) (Table 2). Their EDSS disability scores ranged from 3.0 to 7.0. Peripheral blood mononuclear cells (PBMC) were obtained and tested for their proliferative responses to the myelin peptides by means of a H^3^-Thymidine uptake assay, in the presence or absence of the myelin antigens (listed in Table 2). In addition, each patient's BPMC were stimulated with 0.3 mg/ml PHA, 7.5 Lfu/ml Tetanus toxoid and 20 mg/ml Candida, used as positive control antigens. Patients responding positively to at least one of the myelin peptides (manifested by a S.I. above 2.0) were enrolled to the study; the S.I. was calculated as the ratio of CPM of H^3−^Tymidine uptake in response to a peptide, to the background CPM in the absence of the antigen. T-cell lines were selected according to the proliferation results and prepared for injection. Patients who did not respond to any of the myelin peptides were excluded. One year from the first injection, PBMC were isolated from each patient and tested for reactivity against the 9 myelin peptide epitopes, using the proliferation assay described above.

**Table 2:** Description of the myelin peptides used for the preparation of TCV.

proteins	Peptide name	amino acid location	Sequence
**MBP**	M1	MBP(84–102)	**NPVVHFFKNIVTPRTPPPS**
	M2	MBP(143–168)	**GVDAQGTLSKIFKLGGRDSRSGSPMA**
**MOG**	MO1	MOG(1–22)	**GQFRVIGPRHPIRALVGDEVEL**
	MO2	MOG(34–56)	**GMEVGWYRPPFSRVVHLYRNGKD**
	MO3	MOG(64–86)	**EYRGRTELLKDAIGEGKVTLRIR**
	MO4	MOG(74–96)	**DAIGEGKVTLRIRNVRFSDEGGF**
**PLP**	P1	PLP(41–58)	**GTEKLIETYFSKNYQDYE**
	P2	PLP(184–199)	**QSIAFPSKTSASIGSL**
	P3	PLP(190–209)	**SKTSASIGSLCADARMYGVL**

### 4. Vaccine preparation

Peripheral blood lymphocytes were obtained from each patient and cultured in the presence of a mixture of nine myelin peptides (2 MBP, 4 MOG and 3 PLP) of the myelin proteins, separately. The reactive lymphocytes that showed a stimulation index>2.0 were expanded in vitro into T-cell lines and an individually-tailored vaccine was prepared. Briefly, PBMC obtained from the patients were stimulated with the relevant peptides of the above mentioned myelin proteins (those against which there was a positive proliferative response in each patient) (Table 2). Several rounds of restimulation using irradiated autologous antigen-presenting cells were performed until sufficient numbers of T-cells for four injections were available (around 60×10^6^ cells). The specific T-cell lines were attenuated by irradiation (6000 rads) and injected subcutaneously at 4 time points, on days 1, 60, 90 and 180 (of 10–30×10^6^ cells for each injection). Following the first vaccination, aliquots were frozen for the subsequent injections.

### 5. Quality Control and Assurance (QC and QA)

During T-cell preparation and prior to cell injection, the following quality control tests were performed: mycoplasma, endotoxin, microbiology for gram positive and gram negative growth cultures, cell viability, FACS analysis and cell counting. As part of quality assurance, we also carried out a pre-injection specificity assay using the proliferation assay method (Thy-H^3^). T-cell lines that manifested a S.I. of at least 10 to the relevant myelin protein peptide and no responsiveness to a non-relevant myelin protein peptide mix, were selected for injection. In addition, a precursor frequency assay for each myelin protein was determined (data not shown).

The certified by the Health Ministry, QC/QA controller Shoshanna Frankfurter, Ph.D., from an unrelated laboratory, signed prior to the administration of each injection.

### 6. Statistical analysis

The calculation of the sample size, our assumptions, the randomization process and the justification of the per-protocol analysis only are all explained in section 1 of the Methods.

The analysis of the results was performed by Dr Mario Brass, an independent statistician, using the Wilcoxon Signed Ranks test to evaluate the clinical effects within each group, the Mann-Whitney test for comparing the changes between the groups, and the Fisher's Exact Tests for relapse-related parameters. In addition, the McNemar test, Wilcoxon Signed Ranks test and Chi-Square Goodness of Fit tests were used for the analysis of the MRI data.

## Results

### 1. T-cell reactivities against myelin antigens at study enrollment

Fifty-four relapsing-progressive MS patients were pre-screened for this trial. Thirty two of them (59%) responded to at least one of the above mentioned 9 myelin peptides; 57% responded to MBP peptides, 84% to MOG peptides and 65% to PLP peptides. The 26 patients who responded to at least one of the peptides and who signed the informed consent forms, were enrolled in the study. The patients were randomized and 19 were assigned to the TCV group and 7 to the placebo arm. Twenty-four patients completed the treatment protocol, 17 in the TCV and 7 in the placebo group and were eligible for per-protocol analysis. Two patients were lost to follow up and were excluded from the per-protocol analysis; these patients (numbers 0856 and 8561) had received only 1 or 2 of the 4 programmed injections.

Myelin-specific cell lines were prepared and tailor-made for each patient in the TCV group and the placebo group, as described above. The lines prepared for the patients who were assigned in the sham/placebo group, were frozen. Prior to the injection, the cell lines were tested for their expression of cell surface activation markers as well as for T-cell subset using FACS analysis. Cell lines consisted predominantly of CD4+ T-cells, with a median percentage of 68% CD4+ and 4% CD8+, for the MBP lines, 63% CD4+ and 6% CD8+, for the MOG lines and 57% CD4+ and 23% CD8+, for the PLP lines and had high expression of the CD25 and HLA-DR markers (data not shown).

### 2. Safety of TCV

None of the patients experienced any significant side effect, during the year of follow up. There was a mild and temporary erythema at the site of injection (in the forearm) in 12 out of the 17 patients receiving the TCV and in 2 of the patients receiving the saline injections. No fever, changes in respiratory rate, blood pressure or heart rate were observed. No abnormalities in the white blood counts or blood biochemistry were encountered. No unexpected pathology could be seen in the brain MRI at 12 months following the treatment.

### 3. Effect of TCV on disability (EDSS)

**Figure 2:**
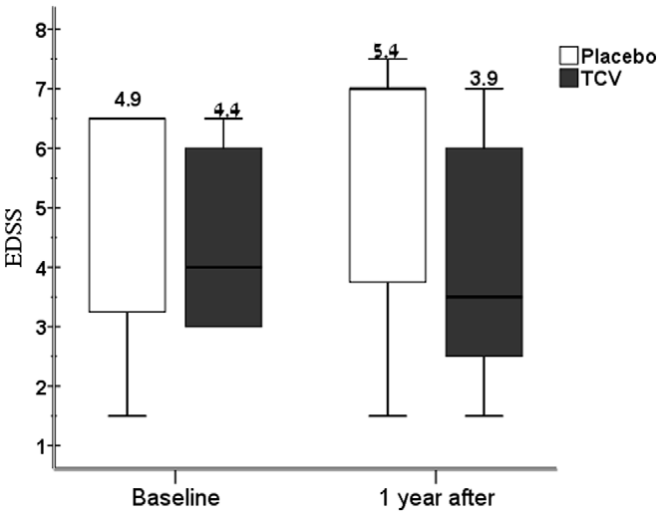
Amelioration of the progression of MS disability, induced by TCV. The mean EDSS scores of the MS patients at baseline (day of inclusion) and at one year after the first vaccination, in the TCV and the sham/placebo group and the change in the EDSS at one year compared to baseline, are shown. Dark barks indicate the vaccinated patients and the blank bars the placebo group.

As part of the clinical follow-up, the EDSS was evaluated by a neurologist blinded to the treatment arm at baseline and at 1, 3, 6, 9 and 12 months. In the TCV group, the mean EDSS decreased (DEDSS = −0.44) at 12 months post-TCV as compared to baseline; in the placebo group, an increase in the mean EDSS was observed at 12 months (DEDSS = +0.5) (Table 3 and Figure 2). The direct comparison of the EDSS changes (at 12 months vs. baseline) between the two groups showed a statistically significant difference favoring the TCV-treated patients (p = 0.01; using the Mann-Whitney test, 2- tailed test). In the TCV group, 8 patients out of 17 exhibited an improvement (decrease in the EDSS) as compared to none in the placebo group. These data indicate a beneficial effect of TCV on MS disability progression.

**Table 3:** Clinical effects of TCV: Effects on disability and relapse parameters during the one-year follow up.

Group	EDSS at baseline	EDSS at 12 months	DEDSS change at 12 months vs. baseline	*P value Exact Sig. (2-tailed) Mann Whitney
**Placebo (n = 7)**	4.86±2.14	5.36±2.38	.50±.41	
**TCV (n = 17)**	4.38±1.54	3.94±2.01	−.44±.90	0.01

	10 M walk at baseline (seconds)	10 M walk at 12 months (seconds)	Change at 12 months vs. baseline	
**Placebo**	6.70±2.12	6.88±1.98	0.18±0.20	
**TCV**	8.60±8.76	7.76±7.58	−0.84±1.44	0.005

	9-hole peg test at baseline (seconds)	9-hole peg test at 12 months (seconds)	Change at 12 months vs. baseline	
**Placebo**	45.01±23.88	47.13±25.83	2.11±3.69	
**TCV**	27.38±10.58	26.48±10.84	−0.91±4.60	0.09

	PASAT at baseline (seconds)	PASAT at 12 months (seconds)	Changes at 12 months vs. baseline	
**Placebo**	37.97±15.63	38.50±17.02	0.53±3.02	
**TCV**	34.41±13.81	40.73±11.79	5.56±10.06	0.22

	Relapses: Proportion of relapse-free patients at baseline	Relapses: Proportion of relapse-free patients during the 12 months of the trial	Reduction in the proportion of patients with ANY relapse during the year of the study in the TCV vs the control group	
**Placebo**	42.9%	42.9%		
**TCV**	35.3%	94.1%	89.6%	**Fisher's Exact Test**: p = 0.01 **Holms adjusted p value**: 0.03

* All statistical comparisons are between the TCV and placebo groups during the year of the trial.

### 4. Effect of TCV on the relapses

**Figure 3:**
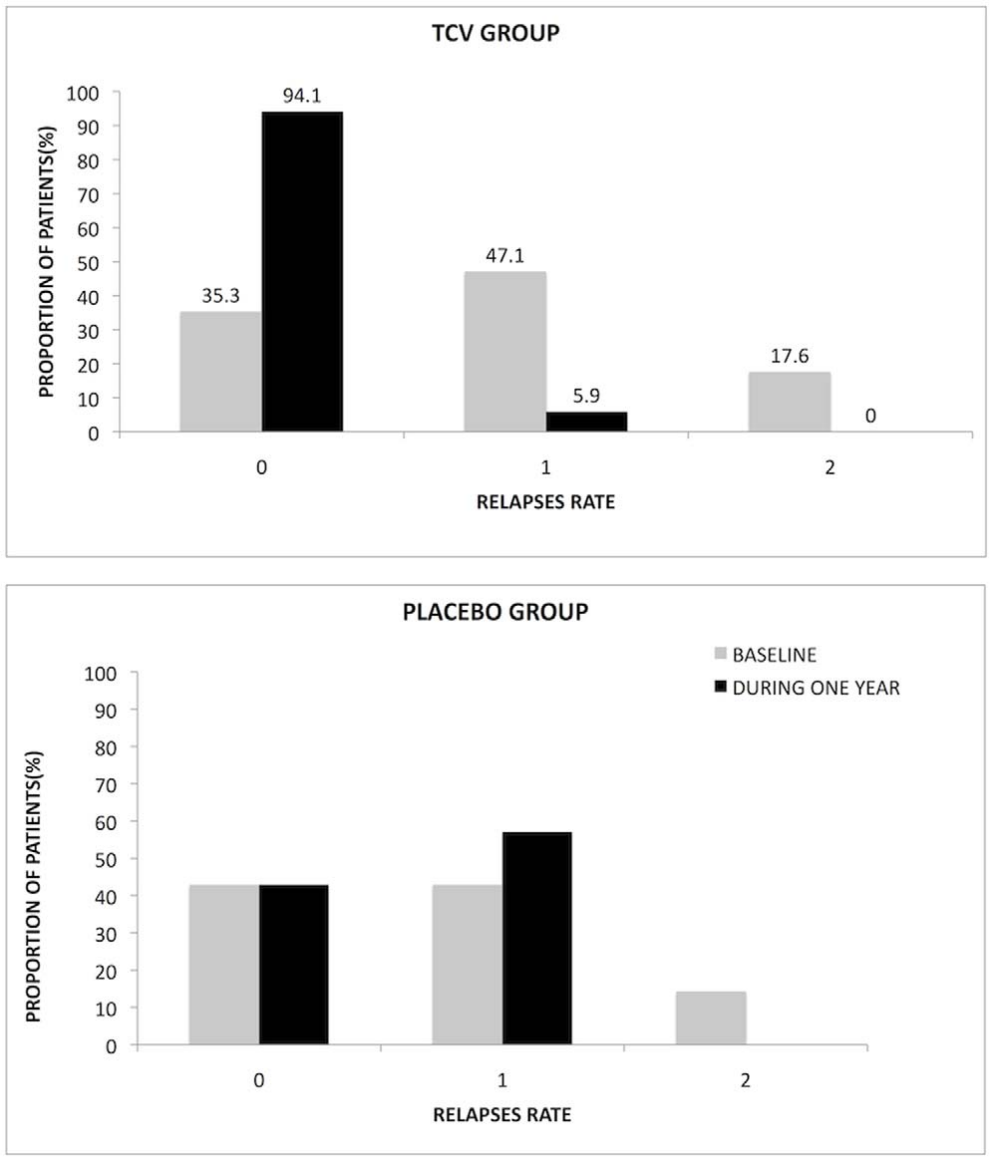
Beneficial effect of TCV on the proportions of patients with relapses. The top figure shows the percentages of patients with 0, 1 or 2 relapses in the TCV group and the bottom figure, the percentages in the placebo group. Gray bars represent the relapses during the year preceding the inclusion and the black bars represent the relapses during the one year following treatment.

Since the numbers of relapses are variable and the control group small, we calculated the numbers and percentages of patients with 0, 1, 2 (or more) relapses (Figure 3) and the proportions of patients who remained relapse-free during the year of the study (Table 3). The relapse rate was reduced in the TCV group during the year following the vaccinations, as compared to the placebo-treated group and to the year prior to treatment. Sixteen out of the 17 patients (94.1%) in the TCV group were relapse-free during the year of the study, as compared to 42.9% in the placebo group (p = 0.01; two-sided Fisher Exact Test and p = 0.03, Holms adjusted for the pre-treatment relapse rates value). Only one relapse was observed in one of the 17 patients in the TCV group, during the year following treatment, as compared to 6 relapses in the 7 placebo-treated patients. The respective total number of relapses in the patients assigned in the TCV-treatment, during the year preceding the inclusion, was fourteen (14). In the placebo group, the percentages of patients with relapses did not change during the year of the study compared to the year prior to inclusion (Table 3 and Figure 3). The reduction in the proportion of patients with any relapse during the year of the study in the TCV vs. the control group, was 89.6% (Table 3).

### 5. Effect of TCV on cognitive function by means of the PASAT test

The PASAT test was used to evaluate cognitive function (especially memory and concentration) changes. The results of each test were recorded as the number of correct answers (mean of 2 trials at each time point) out of 60 (maximal result). In the TCV-treated group, the PASAT score increased (showing improvement) by a mean of 5.56±10.06 at month 12, as compared to the baseline value, whereas in the placebo group there was no change during the study (non-significant increase of 0.53±3.02). The comparison of the changes in the PASAT test between the two groups did not reach statistical significance (p = 0.22, Mann-Whitney test, two- tailed). However, as shown in Table 3, there was a trend of improvement of cognitive function in the TCV group.

### 6. Effect of TCV on the performance in the 9-hole peg test

Recording of the changes in the performance in the 9-hole peg test revealed that in the TCV group, there was a decrease (improvement) of 0.9±4.60 seconds at month 12 as compared to the baseline value, whereas in the control placebo/sham group an increase of 2.11±3.69 seconds, was observed. The comparison of these changes between the two groups revealed a trend of beneficial effect in the TCV group, which did not reach statistical significance (p = 0.09, using the Mann-Whitney, two-tailed test) (Table 3).

### 7. Effect of TCV on the timed 10-meter walking

The walking ability was evaluated by means of the 10-meter timed walking test. At 12 months following the treatment, a decrease in the mean walking time was observed in the TCV group (−0.84±1.44 seconds) and an increase (0.18±0.20 seconds) in the placebo group (Table 3). The comparison of these changes in the walking test between the two groups was highly significant (p = 0.005, Mann-Whitney, two- tailed test).

### 8. Effect of TCV on the lymphocyte reactivities against myelin proteins

**Figure 4:**
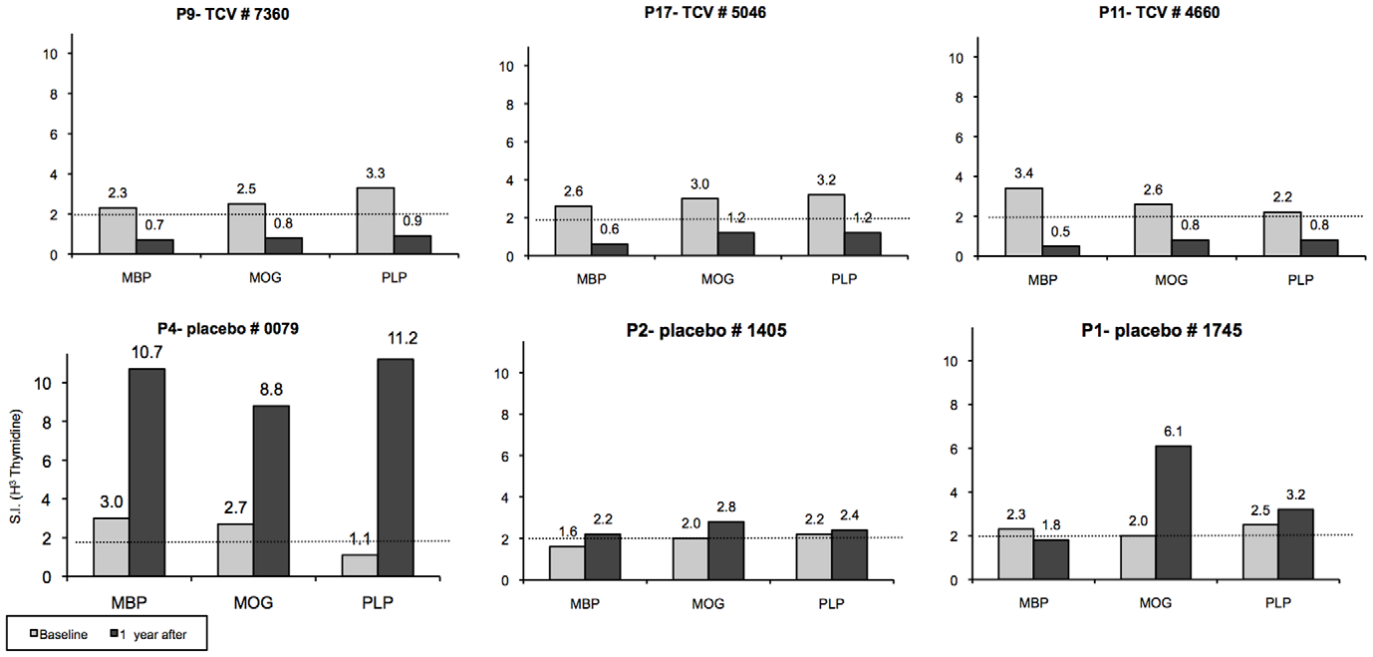
Downregulation of the anti-myelin T-cell responsiveness to MBP, MOG and PLP, following TCV. The peripheral blood lymphocytes' responses to the specific myelin proteins that were used for the preparation of TCV were tested using a proliferation assay (as described in Methods), at 1 year after the treatment (month 12, black bars) and were compared to the baseline (gray bars) values (before TCV). The responses in 3 representative patients from the TCV and the placebo groups are shown.

For the immunological follow-up of the patients in the trial, the responses to the myelin proteins used for the TCV were tested by means of a proliferation assay at 1 year after TCV (month 12) and compared with the baseline values before the treatment. Following one year from the first TCV injection PBMC were isolated and cultured in the presence of the myelin peptides described in the Table 2. Their proliferate responses to the myelin peptides were tested by H^3^- Thymidine uptake assay as described in above. The highest responses to all of the peptides from the same protein were used as a measurement of MBP or MOG or PLP responsiveness. The results are described in Table 4 and Figure 4. In the TCV group, of the nine patients tested after 1 year of TCV, seven showed reduction in the responsiveness to all the myelin proteins tested, patient P3 showed an increase in MOG and PLP and a mild reduction in MBP responsiveness (from a S.I. of 2.7, to 2.1). Patient P15 showed a mild increase in the responsiveness to MBP (from a S.I. of 2.2, to 3.5). In the placebo group, only 3 patients were tested and two showed an increase in their responses to all proteins tested; patient P1 showed a mild decrease in the MBP response (from a S.I. of 2.3 to 1.8) but an increase in MOG- (from a S.I. of 2 to 6.1) and in PLP reactivities (from a S.I. of 1.1, to 11.2). Figure 4 shows the responses against myelin peptides in three representative TCV and 3 placebo patients. Overall, the decrease in responsiveness to the myelin proteins in the TCV group suggests a down regulation of the autoimmune anti-myelin reactivity, induced by TCV.

**Table 4:** Responsiveness to the Myelin Proteins of enrolled patients.

A. TCV Patients						
ID	T- cell proliferationbefore TCV (S.I.)			T-cell proliferation1-year after TCV (S.I.)		
	MBP	MOG	PLP	MBP	MOG	PLP
P1	3	3.8	4.3	nd	nd	nd
P2	1.2	2.4	3	1.2	1.5	1
P3	2.7	1	1.2	2.1	2.5	2.4
P4	1.5	4.2	1.6	nd	nd	nd
P5	1.6	2.7	1.5	nd	nd	nd
P6	3	2.3	2	nd	nd	nd
P7	1.6	4	1.8	nd	nd	nd
P8	2	2.4	1.4	1.4	1.3	1.5
P9	2.3	2.5	3.3	0.7	0.8	0.9
P10	1.7	2.5	2.4	1	2	1.8
P11	3.4	2.6	2.2	0.5	0.8	0.8
P12	1	3.8	1.1	nd	nd	nd
P13	2.3	3	2.7	nd	nd	nd
P14	2	4	2.8	0.8	1.6	1.8
P15	2.2	2	2.4	3.5	2.1	2.4
P16	4.5	5	3.1	nd	nd	nd
P17	2.6	3	3.2	0.6	1.2	1.2
**B. Placebo patients**						
P1	2.3	2	2.5	1.8	6.1	3.2
P2	1.6	2	2.2	2.2	2.8	2.4
P3	7	4.5	2.3	nd	nd	nd
P4	3	2.7	1.1	10.7	8.8	11.2
P5	1.1	2.2	2.8	nd	nd	nd
P6	1.2	1.2	0.8	nd	nd	nd
P7	0.6	0.7	1	nd	nd	nd

nd = not done.

### 9. Effect of TCV on MRI parameters

Brain MRI scans performed with a 1.5 Tesla machine were sent to the Evaluation Center, in San Raffaelle Hospital, Milano, Italy, and were analyzed in a blinded way. The changes in various MRI parameters (total T1 and T2 lesion load, gadolinium enhancing lesion numbers, total lesions volume and normalized brain volume change) from baseline to year 1, did not differ significantly between the two groups (Wilcoxon Signed Ranks test, p values ranging from 0.16 to 0.79, for the various parameters compared) (Table 5).

**Table 5:** Effects of TCV on the MRI parameters.

	At baseline		At the end of the trial (year 1)		
	Placebo group	TCV group	Placebo group	TCV group	P value*
**T2 lesion volume (in ml)**	11.30±0.7	18.82±11.6	11.63±0.7	19.66±11.7	0.79
**T1 lesion volume (in ml)**	2.14±0.7	3.48±3.3	2.82±0.7	3.63±3.6	0.72
**Gadolinium enhancing lesions number**	0.20±0.6	0.63±0.7	0.60±0.7	1.50±2.8	0.52
**Gadolinium enhancing lesions volume (in ml)**	0.06±0.7	0.11±0.2	0.13±0.7	0.20±0.3	0.79
**Normalized brain volume change at year 1 (%)**			0.90±0.7	−0.19±0.6	0.16

* Comparison between the TCV and the placebo group, in terms of change from baseline, using Wilcoxon Signed Ranks test.

## Discussion

We report here the results of a Phase II double-blind study with T cell vaccination (TCV) in progressive MS patients. This is the first double-blind study with TCV in progressive MS (and to our knowledge, the only controlled study in MS, in general), using multiple (4) injections (vaccinations) of attenuated T-cell lines reactive to 9 different myelin peptides. TCV was proven safe and no serious adverse events were documented. There was a profound clinical effect on relapses and a beneficial effect on disability, as evidenced by the follow-up EDSS scores and the performance in the timed 10-meter walking test. The PASAT and the 9-holes peg tests showed a trend for improvement in the TCV group, but this did not reach statistical significance. No significant effects could be detected in the MRI parameters. The discrepancy between the clinical and radiological effects, although somehow puzzling, is not surprising. Physicians are frequently confronted by such discrepancies between the clinical course of MS and the MRI manifestations. Other immunotherapies which were proven clinically effective in MS, showed borderline or no beneficial effects on MRI [12], [13]. The small size of our study may also be one of the reasons for failure to detect a positive effect on MRI parameters. Interestingly, additional TCV studies failed to show significant MRI effects [14], [15], [16], [17], [18], [19].

The mechanisms involved in TCV include induction of anti-idiotypic and anti-ergotypic immune mechanisms. Vaccination with at least 10 million whole attenuated T-cells results in the expansion of a second set of T-cells specific for both idiotypic and ergotypic determinants. Anti-idiotypic T-cells have been shown to consist of both CD4+ and CD8+ T-cells, with fine specificity for hyper-variable TCR regions, including both CDR2 and CDR3 determinants that are likely self-presented by the targeted myelin reactive clones [20]. The CD8+ T-cells may be cytotoxic and/or inhibitory for CD4+ effector T-cells, thus causing deletion or inhibition of pathogenic Th1 T-cell clones present in the vaccinating lines. The CD4+ T-cells are the major cytokine producers, and secrete a variety of cytokines, including IL-4 and IL-10 (Th2 cell phenotype), that may exert regulatory effects on the potentially pathogenic Th1 cells selected for the TCV [15], [21]. Moreover, the regulatory CD4+ subset specific for the vaccinating T-cells may include FoxP3+ Treg cells that inhibit the activation of target T-cells non-specifically, through T-T interactions [22], [23], [24].

The major effect of TCV is to delete or down-regulate the activation of the autoimmune effector T-cells. The disappearance of the T-cell clones that were used in the vaccination procedure has been one of the most consistent findings of the various studies [14] and ours as well. In our trial, in the TCV-treated group, at one year following the treatment, T cell responsiveness to myelin antigens was reduced significantly, as shown in the results of the proliferation assay in three representative TCV patients and three placebo patients (Table 4, Figure 4).

There are seven different clinical trials with TCV at various stages of MS that have been already reported in the literature [14], [15], [16], [17], [18], [19], [25] and six additional studies which are in progress or have been recently completed, but not yet reported (reviewed by Hellings, Raus and Stinissen [10] and more recently by Vandenbark and Abulafia-Lapid [11]). These studies confirmed the consistent reduction of MBP-specific T-cells following TCV and showed some indications of clinical efficacy as documented by the prolongation of the time to MS-progression in both RR-MS and SP-MS patients, as compared to the pre-vaccination rates. More recently, in an open-label TCV study, 20 RR-MS patients were vaccinated with MBP and MOG (panel of peptides) T-cell lines [14]. The results of this study showed that vaccinated patients had a reduction in the annual relapse rate, EDSS stabilization and reduction in MRI activity at one year of follow-up. All the above promising results provided the rationale for our randomized, double-blind clinical TCV trial using T-cell lines selected with MBP, PLP and MOG peptides and multiple injections, in progressive MS patients.

TCV appears to be a very safe procedure, with the attenuation step used in both animal and human studies effectively preventing encephalitogenicity of the injected myelin-reactive T cells. Overall, the previous and our TCV studies lend direct support to the involvement of inflammatory myelin-reactive T-cells in MS pathogenesis strengthening the autoimmunity concept. However, the trends reported in the pilot studies needed to be validated by controlled double-blind, placebo-controlled trials. The present trial provides clear indications of clinical efficacy of TCV on MS. Of importance is the fact that in our study we performed multiple vaccinations and used 9 peptides and a “cocktail” of anti myelin cell lines, which may all account for the more significant efficacy detected in several clinical parameters in our trial.

In conclusion, this blinded clinical trial with TCV in patients with relapsing-progressive MS using anti-myelin cell lines and not T-cell clones, despite its limitations due to the relatively small number of participants and the lack of significant MRI data, shows the feasibility and safety of the procedure and provides indications of clinical efficacy of TCV in MS. Further studies with larger groups of patients and longer follow-up periods, are warranted.

## Supporting Information

Protocol S1 **Trial Protocol.**
(DOC)

Checklist S1 **CONSORT Checklist.**
(DOC)
