# Supplementary material for: T Cell Vaccination Benefits Relapsing Progressive Multiple Sclerosis Patients: A Randomized, Double-Blind Clinical Trial
Source: PLoS One. 2012 Dec 14;7(12):e50478. doi: 10.1371/journal.pone.0050478 (PMC3522721; doi:10.1371/journal.pone.0050478)
Supplement: Protocol S1 — Trial Protocol. (DOC) [file pone.0050478.s001.doc]

**Clinical Study Protocol (03)**

Single site, Randomized, Double-blind, Placebo-Controlled Study to Evaluate the Efficacy, Tolerability and Safety of PLP, MBP, MOG-Specific T-cell Vaccine Subcutaneous Administered in Relapsing Progressive Multiple Sclerosis

Protocol Number: 03

Clinical Phase: Phase I/II

Protocol Version: Version-2

Date: March 10, 2002

Investigators: Dr. R. Abulafia-Lapid

Dr. D. Karussis

Dr. H. Schor

Central Laboratories HADASSAH MEDICAL ORGANIZATION

Department of Nuclear Medicine, HBRC

T-cell Vaccination Unit.

Kiryat Hadassah p.o.b 12000 - 91120 Jerusalem, Israel

Tel: 972-2-6777510 Fax: 972-2-6421203

This confidential document is the property of HBRC/ T-cell Vaccination Center, Hadassah Hebrew University Hospital, Jerusalem. No information contained herein may be disclosed without the prior written approval of the HBRC/ T-cell Vaccination Center. This is a confidential document for the sole information and use of the investigator's team or other authorized entities. Acceptance of this document constitutes the agreement by the recipient that no unpublished information contained herein will be disclosed without the prior written approval of Hadassah Medical Center.

**Table of Contents**

1. **Protocol SYNOPSIS**
2. **ABBREVIATIONS**
3. **INTRODUCTION**
   1. **Network theory of autoimmunity, the basis of T cell vaccination concept and modulation of autoimmunity diseases.**
   2. **Immunomodulation of autoimmune diseases by T-cell vaccination in animal models.**
   3. **Studies in Humans**
   4. **Safety: T cell vaccination subcutaneous administration to multiple sclerosis patients.**
   5. **Rational of conducting the study**
4. **STUDY OBJECTIVES**
   1. **Primary Efficacy Endpoint**

**Primary Safety Endpoint**

- 1. **Secondary Efficacy Endpoint**

**Secondary Safety Endpoints**

- 1. **Tertiary Outcome Measures**

1. **STUDY DESIGN**

**5.1. Overview**

**5.2. Patients Assignment and Treatment Groups**

**5.3. Study Plan**

**5.3.1. Study Location**

1. **STUDY POPULATION**

**6.1. Number of patients**

**6.2. Patient Selection**

**6.2.1 Inclusion Criteria**

**6.2.2 Exclusion Criteria**

1. **Assessments**
   1. **Clinically Definite MS**
   2. **Neurological Evaluations**
   3. **Relapse Evaluation**
      1. **Relapse Evaluation Procedures**
      2. **Relapse Definition**
         1. **Patient Evaluation by the Examining Physician**
         2. **Relapse Determination by the Treating Physician**
         3. **Relapse Treatment**
   4. **MRI Evaluations**
   5. **Adverse Experiences**
   6. **Laboratory Evaluations**
   7. **Other Evaluations**
2. **CONCOMITANT MEDICATIONS**
   1. **Allowed Concomitant Medications**
      1. **Symptomatic MS Medications**
      2. **Cortico-steroid Therapy During Exacerbation**
      3. **Other Allowed Medications**
   2. **Prohibited Medications**
3. **STUDY CONDUCT**
   1. **Pre-Enrollment (Screening) Evaluation (Visit 1)**
      1. **Screening for patients that their T-cells react to the specific peptide**
   2. **Preparation of the T-cell vaccination (visit 2-7)**
   3. **Month 0 Baseline Evaluation – First Day of Treatment (Visit 8)**
      1. **Randomization**
      2. **Post Randomization Procedures**
   4. **Treatment Period (visits 9-11)**
   5. **Follow up period (visits 12, 13)**
   6. **Termination (visit 14)**
   7. **Study Conclusion**
      1. **Completed Patients**
      2. **Premature Discontinuation of Treatment**
         1. **Study Premature Discontinuation Criteria**
         2. **Follow up of Premature Discontinued Patients**
4. **T-CELL VACCINATION SUPLLIES**
   1. **Isolation of PLP, MBP and MOG T-cell lines for the T-cell vaccination**
   2. **Freezing protocol**
   3. **Cell inactivation**
   4. **Characterization of PLP, MBP and MOG-Reactive T-Cell Lines Valuation Prior to the Injection**
   5. **TCV labeling and packaging**
   6. **Storage**
   7. **Quality Control and Assurance of TCV Preparations**
5. **SAFETY/ADVERSE EXPERIENCES/EVENTS**
   1. **Adverse experience reporting**
   2. **The intensity or severity of an AE characterization**
   3. **The relationship and Adverse Event to the study treatment characterization**
   4. **Serious Adverse Events**
6. **Statistical Methodology**
   1. **Randomization Procedure**
7. **ETHICS**
   1. **Informed Consent**
   2. **Ethics Committee (EC)/Institutional Review Board (IRB)**
8. **DOCUMENTATION**
   1. **Site Documents Required for Initiation**
   2. **Additional Site Documents**
   3. **Maintenance and Retention of Records**
      1. **Case Report Forms (CRF)**
   4. **Monitoring**
   5. **Quality Assurance Audits/Inspections**
      1. **Good Clinical Practice**
      2. **Quality Laboratory Standards**
      3. **Quality Assurance Program**

**14.6 Maintaining the Study Blind**

**15. STUDY PERSONNEL**

- 1. **At the Study Site**
     1. **The Treating neurologist /Physician**
     2. **The Examining neurologist /Physician**
     3. **The MRI Radiologist and the MRI Technician**
     4. **The Clinical Coordinator**

1. **REFERENCES**

**Appendices**

**APPENDIX I Relapse Definition**

**APPENDIX II Instructions for Testing Lower Extremity Function: Timed 10 Metter Walk**

**APPENDIX III Ambulation Index**

**APPENDIX IV Magnetic Resoncance Imaging (MRI) Protocol**

1. **Protocol SYNOPSIS**

| **Protocol Number** | 02 |
| --- | --- |
| **Study Title** | Single site, Randomized, Double-blind, Placebo-Controlled Study to Evaluation the Efficacy, Tolerability and Safety of PLP, MBP, MOG peptide-Specific T-cell Vaccine **Subcutaneous** Administered in Relapsing Progressive Multiple Sclerosis |
| **Participating Country** | Israel |
| **Clinical Phase** | Phase I/II |
| **Study Objectives** | To compare the efficacy, tolerability, and safety of PLP, MBP, MOG peptide-specific T-cell Vaccine (10-50 x10^6^ cells) injected subcutaneous four times (day zero and after 30, 90,180 days) vs. placebo. |
| **Study Population** | Relapsing Progressive Multiple Sclerosis patients  R-P MS |
| **Study Design** | Single site, Randomized, Double-blind Study comparing MBP, MOG-peptide Specific T-cell vaccination vs. placebo in Relapsing Progressive Multiple Sclerosis patients.  Eligible patients will be randomized to one of the following groups:   - 10-50 millions dead-autologous cell vaccine. - Matching placebo   Patients will be evaluated in the study center for screening at baseline and then after 1, 3, 6, 8, 10 ,12 month for safety evaluation or neurological exams.  All patients will be MRI monitored at baseline and at study completion.  The data will be monitored by CRO.  The recruitment phase is estimated to be 20 month the treatment and monitoring each patient will be 14 month and the overall duration of the study will be 42 month.  The final analysis will be conducted after all patients complete the 14 month period, and will include all pre-defined end-points. |
| **Number of Patients** | 30 patients |
| **Diagnostic and Main Inclusion Criteria** | Clinically define MS as defined by Poster et al. (Ann. Neurol 1983) disease duration (from onset) at least one year.  R-P MS  Males and female (birth control, where appropriate)  Age: 18-60 years, inclusive.  EDSS at baseline: 3.0-6.5, inclusive.  Evidence of disease progression of 1 degree in the EDSS scale, or at least two severe relapses during the last year prior to inclusion.  MRI of the brain with at least 5 lesions in the white matter (T2 imaging).  Failure of other existing treatments according to the guidelines of the Israeli Ministry of Health.  Patients that their T cells will respond, and be activated by the myelin-derived peptides and a specific T-cell-vaccine can be produce for them.  Ability to give Inform Consent. |
| **Exclusion Criteria** | Patients with other systemic active disease.  Patients who were treated with immunosuppressive drugs/modalities during the 3-6 months (depending on the cytotoxicity of the medication used) prior to the inclusion.  Pregnancy; Pregnant women or women without any efficacious contraception (oral contraception, or intra-uterine device).  Patients with an additional MS-unrelated autoimmune condition or significant allergy.  Patients who cannot fully understand the treatment protocol or are unable to sign the informed consent.  Patients who have previously received cellular immunotherapy.  Patients for whom the clinician thinks that a follow-up period of at least 12 months will not be possible.  Patients who are participating in other experimental protocols. |
| **Route and Dosage Form** | subcutaneous (SC) injections in the arms (1.5-3.0 ml of the dead cell preparation) and matching placebo injections |
| **Treatment Arms** | 10-50x10^6^ PLP, MOG, MBP peptide-specific dead cells, in 1.5-3 ml saline.  Placebo: 1.5-3 ml saline. |
| **Cortico-steroid Therapy During Exacerbation** | Short-term treatment for acute relapses is: fixed dose of I.V. Solumedrol (Methyl-Prednisolon) 1gr/day. No tapering off is allowed or any other schedule or oral administration are not allowed. |
| **Primary Outcome Measures** | The primary efficacy end-point for this study is The ∆ EDSS that will be calculated for each patient by subtracting the beginning and end point EDSS scores. Mean EDSS scores for the two groups (treatment vs. placebo) will be calculated.  The primary safety endpoint for this study are the safety profile of adverse experiences, the tolerability will be based on the number of patients completing the study  The total number of relapses observed during the monitored period. |
| **Secondary Outcome Measures** | Changes from the baseline of clinical parameter; 10 meter walking test, 9-Hole-peg Test, PASAT cognition test and the Ambulatory Index (AI).  The total number of T1-Gd enhancing lesions (in MRI) and of T1 hypo dance black holes.  The volume of T2 lesions: (in MRI) and the degree of brain atrophy i.e. BPF (brain parenchymal factor) width corpus colossus and of lateral ventrical).  Safety profile of clinical laboratory parameters, the vital signs and ECG |
| **Tertiary Outcome Measures** | Proportion of relapse free patients  Time to first relapse.  Proportion of second relapse free patients  Time to second relapse.  Brain atrophy (whole brain).  Number and proportion of activated lymphocytes.  Number and proportion of anti-myelin reactive lymphocytes in the peripheral blood and IgG antibody levels in the cerebrospinal fluid |
| **Statistical Consideration** | Randomization: a total of 30 patients will be randomized into two treatment arms (1:2). 10 patients for the placebo and 20 patients for the vaccination. |

1. **ABBREVIATIONS**

CRF Case report Form

CRO Contract Research Organization

EAE Experimental Allergic Encephalomyelitis

EC Ethics Committee

EDSS Expended Disability Status Scale

FDA Food and Drug Administration

GMP Good Manufactory Practice

IRB Institutional Review Board

MBP Myelin Basic Protein

MOG Myelin Oligodendrocyte Glycoprotein

PLP Proteolipid Protein Peptides

QA Quality Assurance

QC Quality Control

R-P MS Relapsing-Progressive MS

R-R MS Relapsing-Remitting MS

SAE Serious Adverse Event

S.C. Subcutaneous

SOP Standard Operating Procedure

T1 Relaxation Time T1

T2 Relaxation Time T2

TCV T-Cell Vaccination

1. **INTRODUCTION**

**3.1 Network theory of autoimmunity-the basis of T cell vaccination concept and modulation of autoimmunity diseases.**

During the last decade, it has been demonstrated that T-cell vaccination by autoantigen-specific T-cell lines or clones, can inhibit the progression of autoimmune diseases. The term T-cell vaccination was denote the use of attenuated autoimmune T-cells as vaccines to prevent experimental autoimmune diseases (1). It was showed that it is possible to modulate autoimmunity by developing a specific anti-T-cell immune response (2). This work has led to the development of new concepts for the network theory of autoimmunity (3, 4). Schematically, contrary to what was thought in the context of the classical clonal selection theory, it has been shown that specific effector T cells and antibodies against self-antigens can be found under normal conditions in healthy individuals. In the absence of disease, these effector cells are normally inactivated by a regulatory network of interconnected, antigen specific and anti-idiotypic, helper, and suppresser T-cell populations.

According to this view, the occurrence of autoimmune pathology is related to a change in the state of the network from suppression to activation. Such a transition can be experimentally induced by an excess of self-antigen, an excess of activated effector cells, or by a mechanism particularly relevant to the topic of autoimmunity associated with microbial or viral infection, namely by antigens from foreign pathogens which mimic self-antigens (due to structural similarities) (“molecular mimicry”).

Conversely, the state of the network can be altered from activation to suppression by a procedure known as T-cell vaccination, in which autologous effector T-cells are re-injected after *in vitro* stimulation followed by attenuation (*i.e.* killing by ionizing irradiation or cross-linking agents to prevent their proliferation).

**3.2** **Immunomodulation of autoimmune diseases by T-cell vaccination in animal models.**

In the earlier 80^th^ Cohen and Ben Nun have coined the term T-cell vaccination (TCV) after performing a set of experiments. This study suggested that Z1a, a line of T-cell specific for MBP, that was activated with MBP induced experimental autoimmune encephalomyelitis (EAE) in naïve Lewis rates (5). However, a treatment with cells in a single inoculation was sufficient to protect about 70% of rats from subsequent EAE induced actively. Furthermore, activation that was followed by irradiation of this line provided protection against EAE (1). Lines of T lymphocytes reactive against the basic protein of myelin (BP) were found in previous studies to mediate experimental autoimmune encephalomyelitis (EAE) in rats. Holoshitz et al (2) further investigated the effects of T lymphocyte lines reactive to different antigenic determinants on the MBP molecule, such as major encephalitogenic peptide (EP) determinant derived from guinea pig BP (G-BP), and minor, non-EP determinants derived from bovine BP (B-BP). They found that both lines of T lymphocytes could mediate EAE induction. Resistance to active EAE acquired by spontaneous recovery from line mediated EAE or by vaccination with attenuated cells was found to be specific for the particular MBP determinant. This suggests that EAE may be mediated by lines of T lymphocytes reactive to different determinants on the MBP molecule, but the resistance to EAE acquired by exposure to line cells is determinant specific. Hence, acquired resistance to EAE is directed by the receptor specificity.

In a study of the mechanism of resistance to autoimmune disease induced by T-cell vaccination, T-cell vaccination is shown to induce resistance to autoimmune disease by activating an anti-idiotypic network (6). In this study rats, were vaccinated against experimental autoimmune encephalomyelitis (EAE) by injecting them once in the hind footpads with a subencephalitogenic dose (10^4^) of a clone of T lymphocytes specific for myelin basic protein (MBP). The response to vaccination was assayed by challenging the rats with an encephalitogenic dose (3 X 10^6^) of T lymphocytes of this MBP-specific clone. Five to six days after vaccination, the cells responsible for mediating resistance to adoptively transferred EAE were concentrated in the popliteal lymph nodes draining the vaccination site. Transfer of the draining lymph node cells to unvaccinated rats led to loss of resistance in the donor rats and acquisition of resistance by the recipient rats. Limiting-dilution cultures of the draining lymph node cells were established with irradiated cells of the MBP-specific clone as stimulators. Two sets of T lymphocytes specifically responsive to the MBP-specific T-cells from the clone were isolated: CD4+CD8- helper and CD4-CD8+ suppressor cells. The helper T-cells, like the BP antigen, specifically stimulated the BP-specific vaccinating clone. In contrast, the suppressor T-cells specifically suppressed the response of the BP-specific vaccinating clone to its BP antigen (6).

Although myelin basic protein (MBP)-recognizing T-cells are not readily obtained after immunization of BALB/c mice with MBP (reflecting the BALB/c resistance to actively induced experimental autoimmune encephalomyelitis (EAE), they can be expanded and cloned after several rounds of in vitro culture. The majority of BALB/c-derived clones recognize an epitope defined by MBP peptide 59-76. When transferred to naive BALB/c recipients, these clones cause classical EAE, with characteristic inflammation and demyelination of the central nervous system (CNS). It was showed that two related clones recognizing a minor epitope, defined by MBP peptide 151-168, cause inflammation and demyelination preferentially of the Peripheral Nervous System (PNS). Because MBP has alternatively spliced isoforms, residues 151-168 are not present contiguously in all MBP isoforms. In order to determine whether induction of PNS disease is idiosyncratic to these sister clones, or related to their properties of epitope recognition, an independent T-cell line with similar recognition properties was studied. Clone 116F, derived from a BALB/c shiverer mouse, expresses a different T-cell receptor (TCR), with distinct TCR contact residues, but like the previously described T-cells, this clone requires residues from both exons 6 and 7 for optimal stimulation. When adoptively transferred to BALB/c recipients, this clone preferentially induces disease of the PNS. A control BALB/c shiverer-derived MBP 59-76-recognizing clone, in contrast, induces CNS disease. These data strongly suggest that the site of disease initiation may correlate with epitope recognition, particularly when alternative isoforms are involved.

Myelin basic protein (MBP) and proteolipid protein (PLP), the most abundant proteins of central nervous system (CNS) myelin, have been extensively studied as possible primary target antigens in multiple sclerosis (MS), a primary demyelinating autoimmune disease of the CNS. However, there is increasing evidence to suggest that autoimmune reactivity against the quantitatively minor myelin component, myelin oligodendrocyte glycoprotein (MOG), can also play a role in the pathogenicity of MS. It was shown that peripheral blood from patients with multiple sclerosis responds predominantly to myelin oligodendrocyte glycoprotein (MOG), and demonstrated that the immunodominant encephalitogenic epitope inducing chronic experimental autoimmune encephalitis (EAE) in H-2^b^ mice resides within amino acids 35–55 (pMOG 35–55) of the rat MOG sequence. pMOG 35–55 was highly encephalitogenic in several H-2^u^ as well as H-2^b^ mouse strains, but specific clinical expressions were associated with the MHC class II background of the mice (7, 8).

Both strains of mice tested (SJL/J and PL/J mice) were able to mount a primary T-cell response to some of the five MOG peptides synthesized, pMOG 1-21, 35-55, 67-87, 104-117 and 202-218. T-cell lines could be raised in both strains to pMOG 35-55 and pMOG 67-87, but epitope definition revealed that each strain recognized a different minimal epitope within these two peptides. T-cell lines to pMOG 1-21 and pMOG 202-218 could also be raised in SJL/J and PL/J mice, respectively. T-cell reactivity to pMOG 104-117 was not observed in either mouse strain. None of the peptides tested induced detectable clinical signs in SJL/J mice. In contrast, an MS-like chronic relapsing-remitting disease could be induced in PL/J mice with pMOG 35-55 peptide. The disease presented with a delayed onset and with clinical signs that differed significantly in their progression and expression from the typical ascending paralysis of experimental autoimmune encephalomyelitis induced with other myelin components, such as MBP and PLP. Histological examination of CNS tissue from mice injected with pMOG 35-55 revealed only mild neuropathological signs with few inflammatory foci in brain and spinal cord. Some myelin splitting and edema were detected upon electron microscopic examination in the spinal cord and cerebellum. Transfer of pMOG 35-55 reactive T-cells into naive PL/J mice resulted in pathological changes characterized by inflammatory foci in the brain and spinal cord (9). In summary, MOG or MBP major peptides as well as T-cells anti these peptides can induce the disease in animal models.

**3.3 Studies in Humans.**

# Based on data accumulating in animal studies, an initial clinical trial was carried out in four patients with MS by Hafler and co-worker. Patients were inoculated with formaldehyde-fixed autologous T-cell clones isolated by phytohemaglutinin (PHA) stimulation from cerebrospinal fluid. The T-cell clones used for vaccination were chosen based upon their phenotype (CD4+), growth characterization and the expression of dominant, rearranged TCR genes, which represent the oligoclonal T-cell harboring in the brain compartment. The study revealed some interesting immunology findings regarding a general inhibition on T-cell stimulation via the CD2 pathway and increasing autologous mixed lymphocyte responses after vaccination (10).

Based on the successful treatment of EAE by T-cell vaccination in rodents and the potential pathological role of MBP autoreactive T-cells in MS, a phase I clinical trial was conducted by Zhang and co-workers (11). In this clinical trial, ten MS patients were vaccinated with irradiated autologous MBP-specific T-cell clones; MBP-reactive T-cell clones are generated by two-stage procedure within seven weeks. This study aimed to address feasibility toxicity, regulatory T-cell responses and potential changes in the frequency of circulation of MBP- reactive T-cells after vaccination. These preliminary clinical trials suggested that the vaccine clones were well-tolerated without adverse effects. T-cell vaccination was feasible and induced substantial anti-clonotypic T-cell responses to the vaccine clones. The data suggested that inoculation with irradiated MBP- reactive T-cell induces predominant CD8+ T-cell response that coincides reciprocally with a progressive decrease in the frequency of circulating MBP-reactive T-cells in all vaccinated patients. (11). After three inoculations spaced between two to four month, no circulating MBP-reactive T-cells were detected in treated patients, suggesting depletion of the target autoreactive T-cell (the injected cells). These effects were boosted by with each revaccination. Although no clear remission was observed, a series of neurological examinations suggested some clinical improvement regarding moderate reduction of the disease scores (EDSS) and the frequency of relapses in some relapsing remitting patients. Forty percent of the vaccinated patients were clinically stable during the vaccine.

T-cell vaccination protocol was similar to that used in previous clinical studies (11, 12). Briefly, MBP-reactive T-cell clones were pre-activated with 1µg/ml PHA in the presence of irradiated PBMC as a source of accessory cells. Cells were cultured for 5-6 days in RPMI1640 media supplemented with 10% heat-inactivated autologous serum and 50U rIL-2. Activated MBP-reactive T-cells were subsequently washed three times sterile saline. After irradiation (8000 rad, ^60^Co source), cells were washed and resuspended in 2ml sterile saline. Each patient received a total of three injections given S. C. at 2-month intervals. The number of T-cells used for immunization ranged from 40x 10^6^ to 80x 10^6^ cells per injection, which was chosen based on the experience from the previous clinical trial (11).

According to the previous protocol, twenty-seven patients with relapsing-remitting MS were included in this study. Two to four stable MBP-reactive T-cell clones were isolated from each patient. The majority of the T-cell clones obtained recognized the two-immunodominant peptides of MBP residues 83-99 and residues 149-170 or the 111-129 peptides. All clones obtained from a given patient were irradiated and used for immunization (13).

Stnissen, and Raus conducted an extended phase I study in Belgium where forty-nine MS patients were treated with T-cell vaccination (14). The vaccines were generated as described (14). Briefly, autologous MBP reactive T-cells were isolated from the peripheral blood by limiting dilution assays, and subsequently cloned by stimulation with PHA and irradiated feeder cells at low cell densities (2). The MBP reactive T-cells clones were analyzed for their T-cell receptor (TCR) V-(D)-J rearrangements and CDR3 sequence, and were expanded by alternate rounds of stimulation with MBP pulsed autologous PBMC, or irradiated feeders, PHA (1µg/ml) and rIL-2 (5 U/ml). After inactivation by irradiation, 10x 10^6^cells of each clone, with a maximum of six clones, were injected subcutaneously. Three immunizations were performed at 2-month intervals (14).

The results of this study suggested that immunization with attenuated autoreactive T-cells induces a complex cellular response specifically targeted at the vaccine cells, but no antibody responses.

In most patients, MBP-reactive T-cells could not be found over a period of one to three years following vaccination. However, MBP responses reappear during clinical exacerbation. This reappearing MBP-reactive T-cells were epitopes different from those found prior to vaccinations, thus suggesting epitope spreading. Therefore, it is highly probable that MBP-reactive T-cells represents only group of the T-cells responsible for the progression of the disease. There is a need to analyze other anti myelin proteins reactive T-cells.

In a pilot open trial conducted by Weiner’s group (15), they used T-cell lines directed against a broad selection of antigens and not clones. Four secondary progressive MS patients were vaccinated with bovine myelin-reactive irradiated T-cell **lines** from their peripheral blood. Patients were followed for 30-39 months, and monitored for immunological responses toward the vaccine, and for their clinical characteristics. Two patients showed stable EDSS score over time, one patient showed improvement by one EDSS step, and the remaining patient has shown EDSS advanced over time. After the second inoculation, there was a progressive decline of circulating whole myelin-reactive T-cells, MBP143-168, PLP104-117, and MOG43-55-peptide-reactive T-cells. This study shows that vaccination with autologous irradiated bovine myelin-reactive T-cells promotes an effective depletion of T-cells reactive against different myelin antigens (15).

Studies of T-cell reactivity to myelin antigens have led to various and contradictory results. A possible explanation could be that anti-myelin reactivity differs among individual patients due to disease heterogeneity or differences in genetic background (HLA). Furthermore, recent studies indicate that although a single myelin antigen may trigger the onset of MS, the subsequent disease course is accompanied by reactivity to other myelin antigens (16). This so-called “determinant spreading” is most likely a result from ongoing demyelination leading to the release of previously inaccessible myelin components.

In a study conducted in Belgium, T-cell reactivity to MBP, MOG and a panel of synthetic MBP, MOG and PLP peptides in MS patients and healthy subjects were analyzed, using ELISPOT. They did not observe significant differences in reactivity between MS patients and control subjects to any of these myelin antigens and peptides. However, the frequency of MBP-reactive T-cells in IL-2 expanded lymphocytes was analyzed and an increased frequency of MBP-reactive T-cells was found among IL-2 expanded lymphocytes from MS patients as compared with healthy controls. These data further support the view that MBP reactive T-cells are activated in vivo in MS patients. They also analyzed the Th-phenotype of the anti-MBP T-cells in MS and studied whether the cytokine phenotype of these T-cells could be altered in Th1 and Th2 biasing conditions. This study provides further information about the anti-myelin T-cell reactivity in MS.

Although T-cell responses to the quantitatively major myelin proteins, myelin basic protein (MBP) and proteolipid protein (PLP), are likely to be of importance in the course of multiple sclerosis (MS), and cell-mediated autoimmune responses to other myelin antigens. In particular, quantitatively minor myelin antigens, such as myelin-associated glycoprotein (MAG) and the central nervous system-specific myelin oligodendrocyte glycoprotein (MOG), could also play a prevalent role in disease initiation or progression. Highly purified myelin antigens were used in this study to assess cell-mediated immune response to MOG in MS patients, in the context of the reactivity to other myelin antigens, MBP, PLP, and MAG. The greatest incidence of proliferative response by MS peripheral blood lymphocytes was to MOG, as 12 of 24 patients tested reacted and, of these, 8 reacted to MOG exclusively. In contrast, only 1 control individual out of 16 tested, reacted positively to MOG. The incidence, of responses to MBP, PLP, and MAG did not differ greatly between MS patients and control individuals. A predominant T-cell reactivity to MOG in MS suggests an important role for cell-mediated immune response to this antigen in the pathogenesis of MS (17).

Ben-Nun (18) further analyzed another group of 52 MS patients and 49 control individuals for reactivity of their peripheral blood lymphocytes (PBL) to rhMOG and to MBP concomitantly. Of the 52 MS patients tested, 24 responded to MOG and 10 out of 49 responded to MBP, whereas only 5 MOG-reactive and 4 MBP-reactive control individuals were detected out of the 49 tested. These results therefore highly confirm the predominant reactivity to MOG in MS. The analysis of the primary proliferative response to 11 synthetic overlapping peptides (phMOG) spanning the extracellular domain of human MOG by PBL from 9 MS patients and 15 control individuals (9 healthy controls and 6 patients with neurological diseases other than MS), further supports a prevalent role for the autoimmune response to MOG in MS, as only 1 of the 15 controls tested showed reactivity to any of the phMOG, whilst 5 out of the 9 patients studied reacted to at least 1 of the phMOG. PBL from 9 MS patients, and from 4 controls, were selected in vitro with each of the phMOG. Of the 9 patients studied, 7 reacted to at least 1 phMOG upon secondary stimulation and the reactivity was mostly directed to epitopes localized within three main regions (amino acids 1-22, 34-56 and 64-96), as was observed for the primary response of PBL. The predominant response to MOG of PBL from MS patients as demonstrated in two separate studies using native MOG and rhMOG as antigens, and the high incidence of reactivity of these PBL compared to the lack of response to phMOG by control PBL, emphasize the relevance of MOG in MS pathogenesis and support a primary role for the autoimmune T-cell response to MOG in disease development.

In summary, T-cells reactive to MBP, PLP, and MOG are the major population involved in the disease induction and progression. However, these cells can be use in other contexts as vaccine that would abrogate the course of the disease. Applying TCV on MS patients may be a pivotal treatment to cure the disease and a model-treatment for other autoimmune disorders.

**3.4** **Safety: T cell vaccination subcutaneous administration to multiple sclerosis patients.**

Immunization with irradiated autologous myelin specific cells using similar protocol (19) has been shown safe without any significant side effects. Results obtained so far demonstrated that TCV was not associated with serious adverse events as evidenced by standard toxicological tests. Three injections of the suggested amount of irradiated autologous T-cells did not affect the T-cell repertoire and the reactivity against other antigens. Mild side effects previously reported include skin reaction at the injection site, especially after the third immunization; this reflects a local delayed T-cell mediated hypersensitivity response. The possibility of infections due to transmission of infectious agents from the cultured cells is, according to previous reports, very low if at all (19).

**3.5 Rational of conducting the study.**

Rational for conducting the study: the purpose of this study is to develop a T-cell vaccination protocol using autoantigen-specific T-cell lines, and to test its efficacy to inhibit the development of neurological disability in patients with Multiple Sclerosis (MS). Multiple Sclerosis is an autoimmune inflammatory disease mediated by autoreactive T-lymphocytes which attack the proteins (MBP, PLP, MOG, MAG) of myelin sheath, inducing therefore multifocal demyelination and subsequent multi-level central nervous system dysfunction. It is the main cause of chronic (and permanent) neurological disability in the young population (mean age of disease onset~32, mean disease duration~20-30 years). In Israel, there are 3,000-5,000 patients today. The conventional treatments for MS are focused on down-regulation of the autoimmune, myelin-reactive lymphocytes in an attempt to inhibit the continuous (usually relapsing and remitting) demyelination. Corticosteroids and various immunosuppressants (cytotoxic/cytostatic drugs –chemotherapy- and modalities) and immunomodulating (interferons) drugs have been used the last decades for the management of this disease, but unfortunately with limited success and cumulative toxic side effects due to their lack of specificity in their action on immune cells (normal cells are, equally to the autoimmune ones, destroyed by chemotherapy). T-cell vaccination is a new approach which has the advantage of high specificity and low potential toxicity. Apart from its potential therapeutic value, this procedure may provide a new insight to the immunopathogenesis of MS.

1. **Study Objectives**

To assess the efficacy, safety of vaccination with 10-50 millions dead-autologous anti-PLP, anti-MBP and anti-MOG peptides autoreactive T-cells injected subcutaneous four times (at day zero and after 30, 90 and 180 days) to Relapsing Progressive MS patients during 12 month period.

**4.1 Primary Efficacy Endpoint**

Efficacy will be measured by using an "intention to treat" analysis. The establishment of Primary Efficacy Endpoint will be based on the ∆ EDSS will be calculated for each patient by subtracting the beginning and end point EDSS scores. Mean EDSS scores for the two groups (treatment vs. placebo) will be calculated.

The Area Under the Curve (AUC) of the Change from Baseline in EDSS Score: The AUC of the individual changes from baseline in the EDSS score will be calculated suing the trapazoidal rule. The actual dates on which measurements were obtained will be used for this calculation. All EDSS measurements, including those made while the patient experiences a relapse, will be included. This measure will be divided by time on trial to account for variable trial’s exposure periods.

This measure will be tested using baseline-adjusted analysis of covariance. Statistical methods will be used to compare the adjusted means of the two test contrasts incorporating terms from treatment. Baseline EDSS scores will be included in the model as a covariate. The Total Number of Relapses and Relapses Treated with Steroids: Any relapse observed and/or those treated with steroids will be counted and included in this analysis. Statistical analysis will employ the same procedures as described from the primary end-point.

**Primary Safety Endpoint**

The primary safety endpoint for this study are the safety profile of adverse experiences, the tolerability will be based on the number of patients completing the study.

- 1. **Secondary Efficacy Endpoints:**

Changes from the baseline of clinical parameter; 10 meter walking test, 9-Hole-peg Test, PASAT cognition test and the Ambulatory Index (AI).

The total number of T1-Gd enhancing lesions (in MRI) and of T1 hypo dance black holes.

The volume of T2 lesions: (in MRI) and the degree of brain atrophy i.e. BPF (brain parenchymal factor) width corpus colossus and of lateral ventrical).

**Secondary Safety Endpoints:**

Safety profile of: 1. Clinical laboratory parameters

1. Vital sign
2. ECG
   1. **Tertiary Outcome Measures:**

Proportion of relapse free patients

Time to first relapse.

Proportion of second relapse free patients

Time to second relapse.

Brain atrophy (whole brain).

Number and proportion of activated lymphocytes.

Number and proportion of anti-myelin reactive lymphocytes in the peripheral blood and IgG antibody levels in the cerebrospinal fluid

**5. STUDY DESIGN**

**5.1 Overview**

This is a one center, randomized, double-blind, placebo-controlled study comparing 10-50x10^6^ dead cell vaccine versus placebo in relapsing progressive multiple sclerosis patients (in which severe deterioration in the functional status of at least, one degree in the EDSS scale was observed during the last year) for duration of 12 month (active treatment continuation optional).

All patients will undergo MRI at baseline and at termination.

Visit will be conducted at screening (visit 1), randomization (at visit 8) and then one, three, six, eight, ten, and twelve month after visit 8 (the baseline). The vaccination treatment will be administrated in visit 8, 9, 10 and 11. The patients will be further monitored in visits 12 till 14.

Unscheduled visits may be conducted whenever required, such as adverse events and exacerbations.

The patients will be recruited from the Hadassah MS Clinic. The medical staff will include the treating nurse, the treating physician (The one who will perform the regular follow up registration of Adverse Events AE* and evaluation of relapses) and the examining neurologist (the one who will perform the neurological evaluation).

**5.2 Patients Assignment and Treatment Groups**

Patients who meet all eligibility criteria will be randomly assigned in 1:2 ratio to one of the following groups:

- Group A: 10-50x10^6^ dead T-cells vaccine (20 patients)
- Group B: Placebo (sterile normal saline) (10 patients)

**5.3 Study Plan**

After obtaining informed consent, patients will be evaluated for inclusion into the study. Vaccine will be prepared form PLP, MOG, MBP peptide specific autologous T–cells for the appropriate patients. Group A will be treated with the T cell vaccine and group B will be subjected to the placebo. The T-cell vaccine prepared for group B will be properly stored (will be frozen) and will be used after the study is concluded if study shows a beneficial affect for the patients. The effects of immunotherapy will be evaluated on the basis of the clinical, neuroradiological (MRI), and immunological assessments (optional) carried out at inclusion, every three months following the treatment and at the end of the 12-month period of the trial (one year after the first injection).

**5.3.1 Study Location**

The study will be conducted at:

The HBRC (Human Biology Research Center) in the Nuclear Medicine and Medical Biophysics Department.

Vaccine preparation will be performed at The GMP Clean rooms in the Vector Production Facility.

The Neurological Department Hadassah

All are located at Hadassah Hebrew University Hospital, Jerusalem, Israel.

the GMP (good manufacturing practice) clean rooms of the Vector Production Facility Center both at Hadassah Hospital, Jerusalem.

Screening Failure

**Eligible**

**Base line Evaluation***

Clinical Evaluation

MRI Scanning

Laboratory Evaluation

**Not Eligible**

**Screening Success**

**Randomization**

**Placebo**

10 Patients

**T cell vaccination**

(10-50x10^6^dead cells)

20 Patients

**On study evaluation**

**Final Evaluation***

Clinical Evaluation

MRI Scanning

Laboratory Evaluation

**Open labels**

Result analysis

Conclusion

T-cell vaccination of the placebo group-optional

***** As specified in the next table “study task flow sheet”

**Screening Evaluation***

Eligibility Criteria

Signing on informed consent forms

Medical history

Historical and Concomitant Medications

Physical Examination

Neurological Exam and test

Serum β HCG

Inclusion criteria review process

Screening and production of MOG, MBP peptides-responding-T cell vaccine

**Study Activities Flow Chart**

**STUDY TASK FLOW SHEET**

| Procedures | Screening | TCV Processing | Baseline | On Study Evaluation | | | | | |
| --- | --- | --- | --- | --- | --- | --- | --- | --- | --- |
|  | | | TC Injections | | | |  | | |
| *Visits* | 1 | 2-7* | 8 | 9 | 10 | 11 | 12 | 13 | 14 |
| *Months* | *-3* | -2 | 0 | 1 | 3 | 6 | 8 | 10 | 12 |
| Informed Consent | X |  |  |  |  |  |  |  |  |
| Eligibility Criteria | X |  |  |  |  |  |  |  |  |
| Medical History | X |  |  |  |  |  |  |  |  |
| Historical and  Concomitant Medications | X |  | X | X | X | X | X | X | X |
| Physical Examination | X |  |  |  |  |  |  |  | X |
| Neurological Exam and test | X |  | X | X | X | X | X | X | X |
| Laboratory Evaluation/Immunology |  |  | X |  |  | X |  |  | X |
| Serum β HCG | X |  |  |  |  |  |  |  | X |
| MRI-cerebral |  |  | X |  |  |  |  |  | X |
| Randomization |  |  | X |  |  |  |  |  |  |
| TCV |  |  | X | X | X | X |  |  |  |
| Adverse Experiences |  |  | X | X | X | X | X | X | X |

* Weekly visits for blood withdrawals for TCV preparation.

Preparation of T-cell Vaccination

Activity Flow Chart

**Days + visits**

**Protocol**

**PBMC Isolation-**

Ficoll Paque

PLP/MBP/MOG peptide activation

20 µg/ml

(

2x 12 million cells)

**Blood**

-

50 ml heparinized

patient's blood

**Protocol**

day 0

**visit 2**

**Assays**

**Reactivation I-**

7000 rad Irradiated

autologous APC pulsed with PLP,

MBP or MOG peptides

5-20 U/ml rIL-2 (BM)

**Irradiation**

6000 rad

**Preparation for injection**

1

0-50 million cells in 1-3 ml saline

**QC tests**

**:**

2-5 days prior to injection

Microbiology: THIO,TSB

blood agar

micoplasma, endotoxin

viability >70%

Facs analysis

**T-cell Vaccination Protocol for Multiple Sclerosis Patients**

Clinical trial- Phase I/II

QC- Blood Microbiology

**Freeze**

-

10 million/vial

3-15 aliquots

QC- endotoxin

LAL test for PLP/MBP/MOG

peptides, IL-2 and media

additives

day 7

**visit 3**

day 14

**visit 4**

**Reactivation II-IV**

day 17-31

**visits 5-7**

**Subcutaneous Injection**

**Screening-**

3

H

thymidine uptake 3 days assay

expansion of PLP/MBP/MOG lines with

selected peptides

to validate

**PLP, MBP**

**MOG peptide specific T-cell**

**line**

day 35

**visit 8**

Facs analysis

day 0

**visit 1**

**Validation of MBP, PLP**

**MOG responsiveness**

(50 ml patient's blood)

Proliferation assay to PLP,

MBPand MOG peptides

and recall antigens

**Screening-**

3

H

thymidine uptake 3 days assay

expansion of PLP/MBP/MOG positive

wells with

selected peptides

**PLP MBP MOG specificT-cell line**

day 31

visit 7

**visits 9-11**

**3 injections of frozen cells**

**6. STUDY POPULATION**

**6.1 Number of patients**

Thirty Relapsing-Progressive Multiple Sclerosis (R-P MS) patients will be randomized into two groups. The first group will include 10 patients (placebo) and second 20 patients (TCV treated). Drop-out will be replaced. The patients will be recruited from the Hadassah MS clinic.

**6.2 Patient Selection**

Prior to or at the screening visit, all patients will sign and date an Inform Consent Form approved by the Hadassah Helsinki Ethic board and the Israeli Health Ministry Office. Screening will be performed according to the study task flow sheet.

**6.2.1 Inclusion Criteria**

To be eligible for inclusion into the study, each patient must fulfill the following criteria:

1. Clinically definite MS of the relapsing-progressive type (RPMS) according to Poser’s criteria (16).

2. Age; 18-60 years old, inclusive.

3. EDSS: 3.0 to 6.5, inclusive

4. Disease duration: above one year.

5. Evidence of disease progression of 1 degree in the EDSS scale, or at least two severe relapses (needed hospitalization and treatment) during the last year prior to inclusion.

6. MRI of the brain with at least 5 lesions in the white matter (T2 imaging).

7.Failure of other existing treatments according to the guidelines of the Israeli Ministry of Health.

8. All patients will sign the written informed consent form.

9. Males and female (birth control, where appropriate)

10. Patients that their T cells will respond, and be activated by the myelin-derived peptides and a specific T-cell-vaccine can be produce for them.

**6.2.1 Exclusion Criteria**

Patients are to be excluded from the study if any of the following criteria is being fulfilled:

1. Patients with other systemic active disease.

2. Patients who were treated with immunosuppressive drugs/modalities during the 3-6 months (depending on the cytotoxicity of the medication used) prior to the inclusion.

3. Pregnancy; Pregnant women or women without any efficacious contraception (oral contraception, or intra-uterine device).

4. Patients with an additional MS-unrelated autoimmune condition or significant allergy.

5. Patients who cannot fully understand the treatment protocol or are unable to sign the informed consent.

6. Patients who have previously received cellular immunotherapy.

7. Patients for whom the clinician thinks that a follow-up period of at least 12 months will not be possible.

8. Patients who are participating in other experimental protocols.

**7. Assessments**

# 7.1 Clinically Definite MS

A standardized questionnaire will be used to record MS history for each patient. This information will be used by the investigator to determine if the patient meets the inclusion criteria with respect to the existence of history consistent with clinically definite MS as defined by Poser *et al.* (Ann. Neurol. 1983):

- Two attacks and clinical evidence of two separate lesions
- Two attacks; clinical evidence of one lesion and para-clinical evidence of another separate lesion.
  1. **Neurological Evaluations**

Functional Systems status (FS), Ambulation Index (AI), 10 Meter Walking Test, 9-Hole-peg test PASAT cognition test. and Expanded Disability Status Scale (EDSS) will be performed, scores will be assessed based on standardized neurological examination.

**7.3. Relapse Evaluation**

# 7.3.1 Relapse Evaluation Procedures

- Patients will be instructed to telephone their study site immediately should any symptoms suggestive of a relapse appear.
- The patient has to be examined within 7 days of telephone contact as follows:
- The Treating Physician (see Section 15.1.2) will evaluate the patient once any symptom suggestive of a relapse occurs.
- In case of a suggestive relapse during scheduled or unscheduled visit, the Treating Physician will refer the patient to the Examining Physician (see Section 15.1.3).

# 7.3.2 Relapse Definition

A relapse will be defined as the appearance of one or more new neurological abnormalities or the reappearance of one or more previously observed neurological abnormalities. This change in clinical state must last at least 48 hours, and be immediately preceded by a relatively stable or improving neurological state of at least 30 days.

An event is counted as a relapse only when the patient’s symptoms are accompanied by observed objective neurological changes, consistent with an increase of at least 0.5 in the EDSS scores or one grade in the score of two or more of the seven Functional Systems (FS) or two grades in the score of one of the FS as compared to the previous evaluation.

The patient must not be undergoing any acute metabolic changes such as fever or other medical abnormality. A change in bowel/gladder function or in cognitive function must not be entirely responsible for the changes in EDSS or FS scores.

# 7.3.2.1 Patient Evaluation by the Examining Physician

A complete neurological assessment will be performed at each scheduled visit, including: FS, AI, Kurzke EDSS, and 10 Meter Walking Test, 9-Hole-peg test PASAT cognition test.

# 7.3.2.2 Relapse Determination by the Treating Physician

The decision as to whether the neurological change is considered a relapse will be made by the Treating Physician, based on EDSS/FS scores assess bye the Examining Physician.

Follow-up visits to monitor the course of the relapse will be made at the Treating Physician’s discretion, in addition to the assessment at the next scheduled visit, but the neurological assessments will be performed by the Examining Physician.

In case of an ongoing or just started relapse at study termination visit, patients will be followed until the end of the relapse, wherever possible.

**7.3.2.3 Relapse Treatment**

The Treating Physician will make the decision whether or not cortico-steroids should be administered for the treatment of the relapse. A fixed dose of 1g/day of (Methyl Prednisolone Solumedrol) for 5 consecutive days, is the treatment suggested in this protocol.

**7.4 MRI Evaluations**

- All patients will undergo MRI at baseline and at study completion:
  - Total number of T1-Gd enhancing lesions
  - Volume of T1-Gd enhancing lesions
  - Volume of T2 lesions
  - Total number of New T2 lesions
  - Brain atrophy
  - Total number and volume of hypo-intense lesions in T1 scans

**Steroid treatment for relapse will not affect the time schedule of MRI scans.**

All MRI data will be interpreted in a blinded fashion by the MRI Analysis Center (MRI-AC). MIR can be preformed up to 72 hours (3 days) prior to baseline, and within 10 days after the end of the study.

**7.5 Adverse Experiences**

All adverse experiences will be reported and documented as detailed in Section 11 of this Protocol. The relation of adverse experiences to the treatment will be determined according to WHO guidelines.

**7.6. Laboratory Evaluations**

All laboratory testing will be performed in our laboratories at the Hadassah University Hospital in Jerusalem.

-Serum chemistry

- - Glucose
  - Creatinine
  - Calcium
  - Total Bilirubin
  - Urea
  - AST (SGOT)
  - ALT (SGPT)
  - Cholesterol
  - Total protein
  - Albumin
  - Alkaline Phosphates
  - Phosphorous
  - Sodium
  - Potassium

-Hematology

- - Hemoglobin
  - Hematocrit
  - Red Blood Cells (RBC)
  - White Blood Cells + differential count
  - Platelets

-Urinalysis

- - Protein
  - Glucose
  - PH
  - Blood

-Serum β HCG for female patients (at screening visit and termination).

- Immunological (optional) follow up: Immunological follow-up of the patients will include the detection of:

- Frequency of the **specific auto-reactive** T-cells injected one year from the last injection.
- Frequency of activated lymphocytes to recall antigens one year from the last injection.
- Frequency of **anti-myelin reactive** **lymphocytes** (**anti-clonotypic)** in the peripheral blood.
- Anti-idiotypic antibody levels in the peripheral blood.

**7.7 Other Evaluations**

- Complete physical examination, including height and weight
- Vital signs (oral Temperature, pulse, glood pressure) pre- and post-dose;
- Complete inventory of concomitant diseases and medical history;
- Complete inventory of concomitant medications for non-MS conditions in 30 days prior to screening;
- Complete MS history, including:
  - Onset first symptom and date of diagnosis;
  - Number of relapses in the last year prior to study entry;
  - Inventory of concomitant medications for MS symptomatic treatment during one year prior to study entry;
  - Previous MS treatment at any time;
  - Assure documentation of the most recent relapse (records must be maintained in the source documents, as described in section 14.3);
    - Complete neurological examination, including: Functional Systems Status Evaluation
    - Ambulation Index
    - Kurtzke EDSS
    - 10 Meter Walking Test , 9-Hole-peg test PASAT cognition test.
  - ECG.

Side effects will be closely monitored for 7 days after each injection of the vaccine and thereafter during the regular follow-up visits (time points).

Each enrolled patient should inform the treating physician about every symptom occurring within the 7-day period after injection. Physicians in charge will be available of phone calls contact for all period of the study.

Signs and symptoms will be recorded and scored/graded according to the WHO classification.

### Local signs

Site of subcutaneous injection: -> pain: 0=absent; 1=mild (slight discomfort); 2=moderate (resolved by common analgesic therapy); 3=severe (not resolved by analgesic drug, confined to home).

### Systemic symptoms

Fever: 0=absent; 1=mild (less than 38^o^C); 2=moderate (38-39^o^C); 3=severe (over 39^o^C).

Headache: 0=absent; 1=mild (slight discomfort); 2=moderated (resolved by common antalgic therapy); 3=severe (not resolved by common antalgic drugs).

Asthenia: 0=absent; 1=mild (not interfering with normal life); 2=moderate (interfering with normal life but not requiring bed resting); 3=severe.

**8. CONCOMITANT MEDICATIONS**

**8.1 Allowed Concomitant Medications**

**8.1.1 Symptomatic MS Medications**

Symptomatic MS agents, such as anti-cholinergic and spasmolytic drugs are permitted at clinically appropriate doses.

**8.1.2 Cortico-steroid Therapy During Exacerbation**

Short-term (3-7 days) treatment with cortico-steroids will be allowed during acute relapses. The recommended protocol treatment is a fixed dose of I.V. Solumedrol (Methyl-Prednisolon) 1gr/day.

No tapering off is allowed. **Oral steroids are not allowed.**

**8.1.3 Other Allowed Medications**

Other medications not listed in Paragraph 8.2 (below) may be given concomitantly as needed for the patient’s welfare. Administration of al medications, including indication, dose, time, and route of administration, will be recorded in the Case Report Form.

**8.2 Prohibited Medications**

- Interferons;
- Parenteral steroids treatment;
- Oral steroids;
- Chemotherapeutic therapy;
- Immunosuppressive or immuno-modulating drugs;
- IVIg and any other experimental drugs.

**9. STUDY CONDUCT**

The study assessments will be performed according to the study task flow chart.

**9.1 Pre-Enrollment (Screening) Evaluation (Visit 1)**

The patient will be assessed for eligibility criteria by the Treating Physician and neurologically examined by the Examining Physician. Patient’s pre-enrollment must occur within 60 days prior to randomization and the first treatment. Screening evaluation must assure that all inclusion and exclusion criteria have been met.

During the screening visit, the patient must be thoroughly informed about all aspects of the study, including scheduled study visits and activities, and must sign the informed consent.

The following procedures will then be performed:

- Physical examination;
- Vital signs;
- Medical history;
- Historical/concomitant medications;
- History of multiple sclerosis;’
- Neurological evaluation;
- ECG and Chest X-rays;
- Clinical laboratory studies, including:
  - Serum pregnancy test (β HCG) for women;
  - Urinalyses;
  - Hematology;
  - Serum chemistry;
  - Serum samples and lymphocytes for immunological studies.

- Evidence of relapse;

- Inclusion/exclusion criteria;

- - 1. **Screening for patients that their T-cells react to the specific peptide**

Cell preparation and cell proliferation**:** 50 ml of peripheral blood will be drawn from MS patients, supplemented with 10 IU/ml heparin. Peripheral blood mononuclear cells (PBMC) will be isolated by Ficoll Paque (Pharmacia Biotech, Uppsala, Sweden) density centrifugation. Cells will be washed with RPMI with autologous serum. The culture medium contains RPMI supplemented with 1% sodium pyruvate, 1% l-glutamine (200 mM each), gentamicin (20µg/ml) and 2% Hepes (1 M, pH 7.3) (Biological Industries, Kibbutz Beit Haemek, Israel). PBMC will be plated in 96 well round-bottomed micro plates (Falcon, Lincoln Park, NJ., USA) at a cell concentration of 2x10^5^ cells/ well. Each well will contain 100 µl of 2x10^6^ cells/ml and 100µl of medium with twice the recommended antigen concentration. The above assay will be performed in replicates of five. Five wells with cells and medium without antigen will be incubated as a background control. The antigens that will be used in the proliferation assay and their final concentrations are: PHA (Murex Diagnostic Ltd. England) 0.3 µg/ml; tetanus toxoid (Connaught Lab. Inc. Pen. USA) 5 µg/ml; *Candida albicans* (Miles, WA. USA) 20 µg/ml; PPD (Statens Institute, Denmark); synthetic peptides of the human PLP 41-58, 184-199, 190-209, MBP 83-99, 87-110 and 151-170 and MOG 6-26, and 34-56 (obtained from Chiron, produced in a GLP facility that has been approved by the US Institutional Review Boards for use in clinical study identical to this protocol), 5-20 µg/ml. The plates will be incubated at 37^o^C in a 5% CO_2_ humidified incubator. After seven days, cell proliferation will be assayed by ^3^H-thymidine incorporation to DNA determined using scintillation liquid in λ-particle counting (Packard model 2000 counter reader). Radioactive counts per minute (cpm) will be compared between cells cultured with or without the antigens. The proliferation values will be represented as a stimulation index value (S.I.), *i.e*. cpm with antigens divided by the cpm without the antigen (cpm below 300 will not be considered). S.I. values greater than or equal to 2 will be considered to be positive. Positive patients to PLP, MBP and/or MOG will be considered as fulfilling one of the inclusion criteria's.

**9.2 Preparation of TCV (Visits 2-7)**

Patients will visit the clinic weekly for blood withdrawals. Antigen presenting cells (APC's) will be extract from their blood to facilitate the proliferation of specific T-cell lines.

**9.3 Month 0 Baseline Evaluation – First Day of Treatment (Visit 8)**

Prior to randomization, the following will be assessed:

- Changes in medical condition since screening;
- Concomitant medication changed since screening;
- Physical changes since screening;
- Clinical laboratory tests; Routine hematology, blood chemistry, and urinalyses
- Neurological exams including FS, AI, EDSS, 10 Meter Walking Test, 9-Hole-peg test PASAT cognition test.
- Evidence of relapse;
- MRI
- Inclusion/Exclusion criteria confirmation;
- Pre-dose vital signs.

**9.3.1 Randomization**

Randomization will be determined according to a computer-generated randomization list. On the day of the TCV initiation, each patient will be allocated a number in sequential chronological order. The patient will then be assigned a medication according to his/her sequential number. Prior to allocation of patient number, all patients should be identified by their initials, date of birth, and screening number.

The randomization code for each patient will be delivered to the PI (see section15.1.1: treating physician) in individual sealed envelopes for emergency use only. Only in case of a serious adverse event, when the information contained in the envelope is urgently needed for the treatment of the patent, should the investigator open the relevant sealed emergency envelope containing that patient’s treatment allocation. If possible, the CRO should be notified of the event prior to breaking of the code.

On completion of the study, the emergency code envelopes will be collected by the CRO or by the CRO’s authorized representative.

**9.3.2 Post Randomization Procedures**

T-cell vaccination or placebo will be administrated to patients (first treatment at visit 8). The empty tubes will be disposed to biohazard waste without disclosing the labels.

**9.4 Treatment Period (visits 8-11).**

The treatment period will include 4 visits (Visits 8-11), on day 1 (first injection) and months 1, 3, and 6. Following the first injection of the TCV (visit 8) there will be additional 3 boosts of the TCV that will be schedule to month 1, 3, 6 (visits 9, 10, 11). Two subcutaneous (S.C.) injections (1.5-3 ml of dead cell preparation) will be prepared each time: one for each arm and each will contain a mixture of lines specific for one protein. For the schedule of injections and visits, see the following table:

| **Visit** | **Injection #** | **Day #** | **# of Fixed Cells** |
| --- | --- | --- | --- |
| **8** | **1** | **1** | **10-50 x 10^6^** |
| **9** | **2** | **30** | **10-50 x 10^6^** |
| **10** | **3** | **90** | **10-50 x 10^6^** |
| **11** | **4** | **180** | **10-50 x 10^6^** |

In addition the patients will be examined.

- Neurological assessment will be performed at months 1, 3, and 6. The Examining Physician **should not** refer to previous neurological assessments carried out on the patient, prior to the completion of the visit’s neurological assessment;
- Routine hematology, blood chemistry, ad urinalyses will be performed at months 6 (visit 11).
- Documentation and follow-up of each exacerbation will be conducted through the study period.
- Adverse events will be followed carefully at each visit by the Treating Neurologist;

The injection site on the patients arm will be covered by bandage before the examination. Thus, the evolution will not be biased by differences between the placebo and the TCV at injection site.

**9.5 Follow up period (visits 12, 13)**

The follow up period will be (Visits 12-13), on months 8, and 10. The TCV treatment protocol will be performed as follows:

- Neurological assessment will be performed at months 8, and 10,. The Examining Physician **should not** refer to previous neurological assessments carried out on the patient, prior to the completion of the visit’s neurological assessment;
- Documentation and follow-up of each exacerbation will be conducted through the study period.
- Adverse events will be followed carefully at each visit by the Treating Neurologist.

The injection site on the patients arm will be covered by bandage before the examination. Thus, the evolution will not be biased by differences between the placebo and the TCV at injection site.

- 1. **Termination (visit 14)**

The termination visit will include the following procedures:

- Physical examination;

- Vital signs;

- Clinical laboratory evaluation, routine hematology, blood chemistry, and urinalyses will be performed;

- Adverse experiences;

- Concomitant medication;

- Complete neurological examination including FS, AI, EDSS, 10 Meter Walking Test, 9-Hole-peg test, PASAT cognition test.

- Evaluation of relapse;

- MRI

- Termination of double blind phase procedures;

- If an AE is present at the last follow-up visit, it will be followed until the medical condition returns to baseline or is considered stable or chronic

The injection site on the patients arm will be covered by bandage before the examination. Thus, the evolution will not be biased by differences between the placebo and the TCV at injection site.

**9.7 Study Conclusion**

**9.7.1 Completed Patients**

A patient who has met eligibility criteria, has been assigned to a treatment group and has completed 56 weeks of double-blind treatment, is defined as a completed patient

**9.7.2 Premature Discontinuation of Treatment**

The CRO and the local Ethic committee should be contacted by the investigator if a patient prematurely terminates the study.

Premature termination is any withdrawal from the study prior to the scheduled termination visit.

**9.7.2.1 Study Premature Discontinuation Criteria**

- Patient with any clinical intolerable adverse experience/event; **(of grade III or IV) according** to the WHO classification and, in particular, in case of a severe manifestation of hypersensitivity consecutive to a vaccine injection, or of a severe alteration of renal and/or hepatic functions (creatinine clearance lower than 70 ml/min. and ASAT and/or ALAT transaminases higher than 6 times the normal value.
- Patient’s decision to discontinue treatment.
- Initiation of treatment with any other investigational agent;
- Pregnancy’
- Lost to follow-up;
- PI’s decision to prematurely terminate the study for safety or any other reasons.

**9.7.2.2 Follow up of Premature Discontinued Patients**

- Every attempt will made to follow a patient who prematurely discontinued the study until final planned termination visit.
- Any subject who manifests a severe degree of intolerance to the treatment will prematurely discontinued at the discretion of the treating physician, should remain under medical observation as long as deemed appropriate.
- Laboratory tests demonstrating clinical significant changes, should be reported and followed-up until they return to normal or baseline levels. If an AE is present at the last followed-up visit, it will be followed until the medical condition return to baseline or is consider stable or chronic.

1. **T-CELL VACCINATION SUPLLIES**

The T-cells vaccine will be derived form the patient's blood. Each injection will contain specific dead T-cell (10x 10^6^ cells for each specific line) anti PLP, MBP or MOG peptides in 1.5-3 ml saline. For each patient their will be two injection that will be administrated to the patients both arms. The T-cell lines that are specific to peptides derived from MBP will be merged to one injection and the lines that react to MOG or PLP peptides will pooled to the second injection.

**10.1 Isolation of PLP, MBP and MOG peptide T-cell lines for the T-cell vaccination**

PLP, MBP and MOG peptide-reactive T cells will be isolated from the MS patients with the identical methods used for the generation of antigen specific T-cell lines described previously (20). On visit 2, fifty milliliters of peripheral blood will be drawn from MS patients, supplemented with 10 IU/ml heparin. PBMC will be isolated by Ficoll Paque as described above. PBMC will be washed twice with RPMI (GIBCO, USA). The cells will be plated out in 96 well U-bottom plates (Nunc, Roskilde, Denmark) at a density of 100,000 cells per well in the presence of 5-20 µg/ml of PLP, MBP and MOG peptides (Chiron USA or Severn biotech ,Germany). On day 7, 50 ml of peripheral blood will be drawn (visit 3) from MS patients, supplemented with 10 IU/ml heparin. PBMC will be isolated by Ficoll Paque as described above and will be prepared as pulsed APC’s. On day 7, cultures will be restimulated with the autologous irradiated (7,000 rad) PBMC (100,000 cells per well) used as pulsed APC in the presence of the peptides selected, followed by the addition of 5 U/ml rIL-2 (Cetus or Boehringer Manheim approved for human use) after 48 hours. On day 15, all cultures will be tested for their reactivity to the PLP, MBP and MOG peptides in a proliferation assay by measuring the amount of [^3^H]-thymidine incorporation. Positive wells will be considered those in which reactivity to the antigen is at least two times above the background.

## To generate PLP MBP or MOG-specific T-cell lines, positive wells will be collected and restimulated with irradiated autologous PBMC and peptide antigens as described above (visit four). Restimulation will be performed at weekly intervals. PLP, MBP, MOG peptide specific T-cell lines will be restimulated and expanded (visits 5 to 7) to sufficient numbers for 4 injections. The Culture medium

The culture medium will contain RPMI with autologues serum supplemented with 1% Sodium-Pyruvate, 1% L-Glutamine (200 mM Seromed, Berlin Germany), ), gentamicin. (20µg/ml)

10.2 Freezing protocol

Aliquots of ten million cells per 1 ml freezing media (Cosmic calf serum (HYCLONE) certified for human use and 10% DMSO) will be freezed (-190°C in liquid nitrogen) and stored for future injections.

**10.3 Cell inactivation**

One day prior to the injection date, if indicated by the results of functional tests, the cells will be washed and incubated in culture medium at 10-15 x 106 cells/ml irradiated at 6,000 RAD and then resuspended in 1 ml of PBS or saline and stored at 4^o^C.

10.4 The following characteristics of these PLP, MBP and MOG peptide-reactive T-cell lines will be evaluated prior to the injection:

1. Expression of TCR ab or TCR-gd and CD4 or CD8 by flow cytometry.

2. The fine specificity of the T-cell lines will be examined in a proliferation assay (18), including PLP, MBP and MOG peptides as described above (see screening procedure).

3. Determination of cytokine profile; the presence of IL-2, IL-4, IL-10, IFN-g and TNF-a in the supernatants of unstimulated and antigen-activated (with PLP, MBP and MOG peptides) will be analyzed by sandwich ELISA (19).

4. Quality Control (QC) tests for microbiological, mycoplasma and endotoxin contaminations will be performed 2-3 days prior to injection.

**10.5 TCV labeling and packaging**

The TCV and the placebo will be packed in identical tubes that are covered to prevent distinguishing between the two groups. The label on the tube will specify the patient number and date.

**10.6 Storage**

The T-cell lines will be stored in liquid nitrogen-190°C and thaw, washed and irradiate one day prior to administration day. The dead T-cell will be stored in appropriate tubes at 4^o^C.

**10.7 Quality Control and Assurance of TCV Preparations**

A. Microbiology tests: two days prior to the cell injection, negative and positive gram stain assays will be performed and broth culture done to detect contaminations. Bacteriological contaminations will be tested using TSB (soya been broth test) for aerobic and TIOH (thio-glycolate broth test) for anaerobic. Agar blood will be used only for positive TSB and/or TIOH tests. The microbiology test will be performed at Hadassah Hospital in the Microbiology laboratory.

B. Mycoplasma and endotoxin: Contaminations for mycoplasma and endotoxin will be tested three days prior to the cell injection. In addition, a viability test will be performed before the cell irradiated and only cultures containing 50% or greater viable cells will be used for treatment.

C. Validation of the irradiated cells or viability: validation studies of the radiation source and the viability of the PBMC's will be performed to establish the performance of the irradiator. The cobalt^60^-radiation device at the Radiation Unit in Hadassah will be validated once a year. In addition, T-cell viability test will be performed once in three month starting from the beginning of the study. The cell viability will be study by monitoring the proliferation competence of the cell in response to mitogenic stimuli *in vitro*. Specifically, peripheral Blood Mononuclear Cells (PBMC’s) will be purified from a healthy-donor blood using Ficoll gradient. Ten million cells in one milliliter will be irradiated with 2000 rad, 4000 rad, 6000 rad or 8000 rad. The irradiated cells will be cultured (2x10^6^ cells/ml) in RPMI supplemented with 10% autologous serum and 0.3µg/ml PHA for 24 hours. The cells viability will be monitor by H^3^ -Thymidine uptake assay for 4 hours. This assay measures the incorporation of thymidine into the cell's DNA as result of cell division and DNA replication. Furthermore, viability frequency of the irradiated cell will measured by flow cytometry analysis (FACS) using propidium iodide (PI) staining. This procedure takes advantage of the FACS FL2 detector to recognize dead cells that incorporate the red fluorochrome propidium iodide into cellular DNA. Briefly, Irradiated cells (1x10^5^) will be treated with PI (1µg/ml) and immediately will be analyze by FACScan (Becton Dickinson). The frequency of dead cell will be determined.

D. In our facilities, special care has been taken to prevent contaminations (GMP facility for the T-cell cultures). QC microbiological tests such as TSB and TIOH broth cultures, endotoxin, and mycoplasma tests will be performed prior to the injections. All peptides (Severn Biotech) would be made to a high purity of in excess of 95% purity, typically 99% by HPLC and verified by Mass spectrometry. (MALDi-TOF) and follow ISO9000 principles of manufacturing. Materials (Citus for r-IL2) will be GLP-approved for human injection. Culture media will be serum-free or will contain autologous serum.

**11. SAFETY/ADVERSE EXPERIENCES/EVENTS**

**11.1 Adverse experience reporting**

The investigator must report all adverse experiences (AEs) that occur throughout the study according to the appropriate procedures listed below. In case the investigator becomes aware of any possibly Serious Adverse Event (SAE), including death, he must report this event by phone to the CRO and the local Ethic Committee within 24 hours of notification of this event.

**11.2 The intensity or severity of an AE is characterized as:**

- **Mild**: AE which is easily tolerated.
- **Moderate**: AE sufficiently discomforting to interfere with daily activity.
- **Severe**: AE that prevents normal daily activities.

**11.3 The relationship and Adverse Event to the study treatment is characterized as follows:**

| **TERM** | **DEFINITION** | **CLARIFICATION** |
| --- | --- | --- |
| **Unrelated** | This category applies to those adverse events that, after careful consideration, are clearly and incontrovertibly due to extraneous causes (disease, environment, *etc*.) |  |
| **Unlikely** | In general, this category can be considered applicable to those adverse events that, after careful medical consideration at the time they are evaluated, are judged to be unrelated to the TCV. | Ad adverse event may be considered unlikely to be related if or when two or more of the following are found:   - It does not follow a reasonable temporal sequence from the TCV; - It could readily have been produced by the subject’s clinical state, environmental, or toxic factors, or other modes of therapy administered to the subject; - It does not follow a known pattern of response to the TCV; - It does not reappear or worsen when the TCV is re-administered. |
| **Possibly** | This category applies to those adverse events for which, after careful medical consideration at the time they are evaluated, a connection with the TCV appears unlikely but cannot be ruled out with certainty. | An adverse event may be considered possibly related if or when two or more of the following are found:   - It follows a reasonable temporal sequence from the start of the TCV; - It could not be reasonable explained by the known characteristics of the subject’s clinical state, environmental or toxic factors or other modes of therapy administered to the subject; - It disappears or decreases on cessation of TCV. There are important exceptions when an adverse event does not disappear upon discontinuation of the TCV, yet therapy-relatedness clearly exists (*e.g.* bone marrow depression, fixed drug eruptions. - It follows a known pattern of response to the TCV. |

**11.4 Serious Adverse Events**

Definition: A Serious Adverse Event (SAE) is defined as any adverse experience occurring at any TCV injection:

- Results in death;
- Is life threatening;
- Requires inpatient hospitalization or prolongation of existing hospitalization;
- Results in a persistent or significant disability;
- Is a congenital anomaly/birth defect;

Medical judgment should be exercised in deciding whether expedited reporting is appropriate in other situations, such as important medical events that may not be immediately life-threatening, result in death, or result in hospitalization, but may jeopardize the patient and/or may require intervention to prevent on of the other outcomes listed in the definition above. These could also be considered as serious.

Any SAE, whether deemed TCV related or not, must be reported immediately to the EC and the CRO.

**12. Statistical Methodology**

A total of 30 patients equally randomized to treatment with T-Cell vaccination (TCV) 10-50x10^6^ irradiated cells, (20 patients), or to placebo, (10 patients), are expected to be recruited into this double-blind, placebo controlled. The trail is planned for 4 injection of double-blind treatment. Since the recruitment phase will last for 20 months, two interim analyses are planned to be conducted utilizing 30% and 80% of the accumulated trail information.

**12.1 Randomization Procedure**

After a patient meets the eligibility criteria, he/she will be allocated to one of the 2 treatment groups, based on randomization procedure employing a 2:1 assignment ratio, and a scheme using blocks stratified by centers. The randomization scheme will be prepared by the Sponsor's Statistics & Data Management (S&DM) Department, using the SAS® random number procedure.

The randomization list and the seed used to generate it will be kept sealed in the fire-protected safe.

Once the enrollment of the patient is approved, the enrolling investigator will assign the patient an ID number (patient number) according to the chronological order in which patients are randomized. Patients dropping out from the study after randomization will not be replaced.

Any difficulties in providing TCV treatment to the patient, such as cells thawing problems, low cell number, lack of cell for the vaccination, errors in patient numbers or other anomalies, should be reported immediately to the LCTM who will notify the S&DM Department and other individuals for whom this information is relevant.

**12.2 Significance levels**

The overall significance level for this study will be 5% using two-tailed tests. The treatment effect of the oral glatiramer acetate will be tested for significance by performing two comparisons (contrasts) for each end-point: the group treated with TCV will be compared to placebo. Hochberg's step-up modification to Bonferroni's method will be used to maintain the experiment-wise type I error.

**12.3 Sample Size Rationale**

The primary end-point used for sample size assessment is the total number of relapses observed during the 1-year of double-blind treatment. The power of the trail was estimated based on simulations designed according to the following assumptions:

- An untreated patient (placebo) was assumed to have a relapse rate of λ_i_ at which relapses occur. For each patient the value λ_i_ was randomly selected from an exponential distribution with θ = Σλi/n. this approach represents the use of the Negative Binomial distribution with r = 1. According to sponsor's previous experience in three double-blind placebo-controlled clinical trails in R-R MS patients, the expected placebo relapse rate for 1-year study (θ) is 0.90 relapses/year. Note that this estimate was based on ITT estimates, which are corrected for anticipated withdrawals.
- The expected reduction in relapse rate compared to placebo is 10% for one glatirmer acetate arm and 30% for the other active arm.
- The final analysis will be preformed at the end of the 1-year period of the double-blind treatment as two interim analyses are planned for the trail, the alpha level left for the final analysis is 0.0428179. this` alpha level was used for power estimation.
- The principle statistical analysis is based on the outcome of contrasts (TCV vs. placebo) derived from the Quasi-Likelihood (over dispersed) Poisson Regression (SAS® PROC GENMOD). Adjustments for multiple comparisons were made according to Hochberg's modification to Bonferroni's method.
- All analyses will be based on two-tailed tests..
  1. **Analysis coherent and Handling of Missing Data**

Tree data cohorts are define for this clinical trial. These cohorts are:

- Intent-to-treat Cohort (ITT): consists of all patients who have been randomized. In accordance with the ITT principles, all patients randomized will be kept in their originally assigned treatment group.
- Completers Cohort: Consists of all patients who completed the 12 month of double-blind treatment.
- Evaluable Cohort: Consists of all patients who completed the 12 month of double-blind treatment and did not have any major protocol violations.

For the ITT cohort, the Last Observed Carried Forward (LOCF) approach will be applied, when deemed appropriate, to account for missing data at or prior to study termination (month 12)

The MRI data analyses will also be based on two patient cohorts:

- The MRI Completers Cohort: Consists of all patients having both basline and termination visit scans
  1. **Comparability of Treatment Group at Baseline**

The two treatment groups will be compared for baseline characteristics in each of the above-mentioned cohorts. This analysis will include general medical history, physical examinations taken prior to trial treatment initiation, baseline laboratory data and baseline MS disease measures. The continuous variables will be examined using one-way analysis of variance or the Kruskall-Wallis test, when appropriate, and the categorical variables will be examined for differences between group using the chi-Square test or Fisher's Exact test, when appropriate.

**13. ETHICS**

**13.1 Informed Consent**

The principles of Informed Consent, according to ICH -GCP guidelines, will be followed. A subject should not enter a clinical study until he/she has been properly informed and has freely given his/her consent by signing and dating two (2) original copies of the Informed Consent Form prior to performing any study related procedures.

One original copy will be given to the subject and the second one will be maintained at the site, following the signature and dating by the investigator and/or witness (where appropriate).

A copy of the proposed consent form must be submitted to the Ethics Committee (EC), together with the protocol, for approval.

**13.2 Ethics Committee (EC)/Institutional Review Board (IRB)**

An Ethics Committee/ must approve the study in writing. A copy of the Approval from the EC/IRB and the hospital director’s approval letter, which also contains specific identification of the documents approved, must be received prior to site initiation.

Any amendments to the protocol or subsequent changes to the informed Consent as a result of changes to the protocol and/or investigator brochure must be approved by the EC/IRB and documentation of this approval. Records of the EC/IRB review and approval of all documents pertaining to this study must be kept on file by the investigator and are subject to Regulatory Authorities inspection during or after completion of the study. Unexpected and associated SAEs must also be reported to the EC.

Periodic status reports must be submitted to the EC as required, as well as notification of completion of the study and a final report where applicable.

**14. DOCUMENTATION**

**14.1 Site Documents Required for Initiation**

Prior to the initiation of the study, the following items must be received

1. A signed copy of the protocol, amendments, and notifications (if applicable);
2. EC Written approval for the Protocol, Amendments, Informed Consent, Patient Information Sheet (if applicable), Advertisements (if applicable),
3. The investigator’s curriculum vitae, as well as the curriculum vitae of any co-investigator(s);
4. A list of the persons involved with the study and their signatures;
5. Study site agreement(s);
6. EC Membership list and EC operation procedures
7. Health Regulatory approval.

**14.2 Additional Site Documents**

Prior to the initiation of the study, the following items will be prepared:

- Study Protocol
- Investigator’s Brochure;
- Case Report Forms;
- Study Manual (Protocol SOPs for study specific procedures) and CRO’s SOPs.
- Model of Informed Consent;
- Central Laboratory Certification and Normal Ranges;
- Patients Information Leaflet;
- Insurance Certificate.

**14.3 Maintenance and Retention of Records**

It is the responsibility of the investigator to maintain a comprehensive and centralized filing system of all relevant documentation.

**14.3.1 Case Report Forms (CRF)**

CRFs for individual subjects will be provided by a professional CRF design company and or with cooperation with the CRO. CRFs are used to record study data and are an integral part of the study and subsequent reports. Therefore, the CRFs must be legible and complete. CRFs for this study will be maintained in a study binder and will be reported on a non-carbon (NCR) paper in the CRF (3 copies). One copy will be kept by the investigator and the two other copies will be collected by the study monitor. All forms should be complete using a dark ball point pen to ensure the data copies onto all CRF pages. Errors should be lined out *but not obliterated*, and the correction inserted, initialed, and dated by designated study personnel. Further data corrections will be performed on special “Data Clarification and Resolution Forms” (DCRF). Such DCRF will be dispatched to the investigator in case of erroneous or unclear data. After the correction is made by the investigator on the DCRF, a copy will be kept in the CRF file and the original given to the study monitor.

A CRF must be completed and signed by the relevant investigator (*i.e.*, neurological evaluation by the Examining Neurologist) for each subject enrolled, including those removed from the study for any reason. The reason for removal should be noted on the study conclusion CRF by the investigator for each subject.

CRFs must be kept current to reflect the subject’s status at each phase during the course of the study. Subjects are not to be identified on CRFs by name; appropriately coded identification and the subject’s initials must be used. The investigator must keep a separate log of the subjects’ names and addresses.

Source documents such as originals of laboratory and other test results are to be maintained separately from the CRF in order to allow data verification.

**14.4 Monitoring**

The CRO will be responsible for coordinating the activities of the study monitoring team, to ensure adherence to the study protocol, ICH-GCP guidelines and the CRO’s standard operating procedures. Study Monitors for this trial will be provided by the CRO. The monitors will act in compliance with ICH Guidelines.

**14.5 Quality Assurance Audits/Inspections**

**14.5.1 Good Clinical Practice**

The study described in this protocol will be carried out according to internationally accepted standards of Good Clinical Practice. All procedures not described in this protocol will be performed according to written standard Operating procedures unless otherwise justified.

**14.5.2 Quality Laboratory Standards**

Laboratory tests/evaluations of study described in this protocol will be conducted in accordance with quality laboratory standards and GLP (Good Laboratory Practice).

**14.5.3 Quality Assurance Program**

The study progress will be inspected according to the CRO’s QA inspection program. The final report will be audited to ensure that, as far as can reasonable be established, the methods described and the results reported accurately reflect the raw data generated during the study.

The purpose of such audit will be to determine whether or not the study is being conducted and monitored in compliance with recognized GCP guidelines or laws. An initial review will increase the likelihood that the study data and all other study documentation can withstand a subsequent inspection by any regulatory authority.

Such audits will be arranged within a reasonable time frame and agreed through the monitor.

**14.6 Maintaining the Study Blind**

The investigators (both the treating and examining neurologists, nurse coordinators, pharmacist and/or their staff at the study site), MRI personnel and the personnel involved in patient assessment, monitoring analysis and data management (excluding the personnel of the T-cell Process Laboratory), are blinded to the patient assignment. In order to ensure that information, which could potentially bias handling of data, is not disclosed, the following precautions are practiced:

- Randomization code envelopes are maintained by the PI in a protected file. No access will be permitted to those involved in patient data handling;
- Patients’ general medical evaluation will be assessed separately from their neurological assessment evaluation by two different neurologists. An examining neurologist will assess the patient’s neurological examination, unaware of the patient’s well being, and a testing neurologist will assess whether a patient experiences a relapse and will prescribe steroids or other concomitant medications as needed.

**15. STUDY PERSONNEL**

**15.1 At the Study Site**

At the study center the staff will consist of a minimum of two neurologists/physicians (a treating neurologist and an examining neurologist), MRI radiologist and technician, and a clinical coordinator (who can be a nurse or a neurologist). Treating neurologist will be the Principal Investigator.

**15.1.1 The Treating neurologist /Physician**

The treating neurologist will be responsible for patient eligibility evaluation, the physical examination, the supervision of the TCV treatment regimen, the recording and treating of adverse experiences, the monitoring of safety assessments, including routine laboratory parameters, and coordinating MRI performance.

He will determine if a patient experiences a relapse and whether to treat his relapse or not.

**15.1.2 The Examining neurologist /Physician**

The examining neurologist will be responsible for conduct the neurological examination (EDSS)

**NOTE: It is particularly important that the Treating Physician and the Examining Physician will not discuss safety issues together. The Examining Neurologist will not ask the patient any questions regarding his well being.**

**Both the Treating Physician and the patient will be informed of the importance of not discussing safety issues with the Examining Physician.**

**15.1.3 The MRI Radiologist and the MRI Technician**

The radiologist will be responsible for patient’s capability to undergo MRI scans, and evaluation of the MRI results. The MRI technician will perform of the MRI scans according to the protocol guidelines during the study, the preparation of the hardcopies and the electronic material for evaluation, as well as providing adequate storage and backups of the primary data at the site.

**15.1.4 The Clinical Coordinator**

A nurse or a physician will be responsible for patient scheduling and completing of all patients’ case reports forms.

S/He will instruct the patient concerning the protocol details of the TCV S/He will collect and forward blood samples and requests to the appropriate laboratories, will obtain and forward laboratory results and will assist all site study staff. S/He will also perform the additional clinical tests (10 Meter Walking Test, 9-Hole-peg test, PASAT cognition test.).

**16. REFERANCES**

1. Ben-Nun A, Wekerle H, Cohen IR. Vaccination against autoimmune encephalomyelitis with T-lymphocytes cell lines reactive against myelin basic protein. Nature 1981:292, 60-1.
2. Holoshitz J, Freckle A, Ben-Nun A, Cohen IR. Autoimmune EAE mediated or prevented by T-lymphocyte line directed against diverse antigenic determinants of myelin basic protein. Vaccination is determinant-specific. J. Immunol. 1983, 131,2810-13.
3. Cohen IR, Regulation of autoimmune disease, physiological and therapeutic. Immunol. Rev. 1986,94, 5-21.
4. Cohen IR, and Atlan H. Network regulation of autoimmunity: An automaton model. J. Autoimmun. 1989, 2, 613-25.
5. Ben-Nun A, Cohen IR.Vaccination against autoimmune encephalomyelitis (EAE): attenuated autoimmune T lymphocytes confer resistance to induction of active EAE but not to EAE mediated by the intact T lymphocyte line. *Eur J Immunol*. 1981 Nov; 11 (11): 949-52.
6. Lider O, Reshef T, Beraud E, Ben-Nun A, Cohen IR. Anti-idiotypic network induced by T-cell vaccination against experimental autoimmune encephalomyelitis. *Science*. 1988 Jan 8; 239(4836): 181-3.
7. Mendel I., Kerlero de Rosbo N., Ben-Nun A. 1995. A myelin oligodendrocyte glycoprotein (MOG) peptide induces typical experimental autoimmune encephalomyelitis in H-2^b^ mice. Fine specificity and T-cell receptor Vb expression of encephalitogenic T-cells. *Eur. J. Immunol.* 25: 1951–9.
8. Mendel I., Kerlero de Rosbo N., Ben-Nun A. 1996. Delineation of the minimal encephalitogenic epitope within the immunodominant region of myelin oligodendrocyte glycoprotein. Diverse V‚ gene usage by T-cells recognizing the core epitope encephalitogenic for TCR-V‚b and TCR-V‚a H-2b mice. *Eur. J. Immunol.* 26: 2470–9.
9. Kerlero de Rosbo N., Mendel I., Ben-Nun A. 1995. Chronic relapsing experimental autoimmune encephalomyelitis with a delayed onset and atypical clinical course, induced in PL/J mice by oilgodendrocyte glycoprotein (MOG)-derived peptide: Preliminary analysis of MOG T-cell epitopes. *Eur. J. Immunol.* 25: 985–93.
10. Hafler DA, Cohen IR, Benjamin DS, Weiner HL. T-cell vaccination in multiple sclerosis; a preliminary report. Clin. Immunopathol. 1992, 62, 307-17.
11. Zhang J, Medaer R, Stinissen P, Hafler D, Raus J. MHC-restricted depletion of human myelin basic protein-reactive T-cells by T-cell vaccination. Science 1993, 261, 1451-54
12. Medaer R, Stinissen P, Truyen L, Raus J, Zhang J. Depletion of myelin-basic-protein autoreactive T-cells by T-cell vaccination: pilot trial in multiple sclerosis. Lancet. 1995 Sep 23;346(8978):807-8.
13. Zang YC, Hong J, Tejada-Simon MV, Li S, Rivera VM, Killian JM, Zhang JZ. Th2 immune regulation induced by T-cell vaccination in patients with multiple sclerosis. Eur J Immunol. 2000 Mar; 30(3):908-13.
14. Stinissen P, Medear R, Raus J, Preliminary data of an extended open label phase. 1999 J Neuroimmunol .90: A564
15. Correale J, Lund B, McMillan M, Ko DY, McCarthy K, Weiner LP. T-cell vaccination in secondary progressive multiple sclerosis. J. Neuroimmunol. 2000 Jul 24;107(2):130-9.
16. Correale J, Lund B, McMillan M, Ko DY, McCarthy K, Weiner LP. T-cell vaccination in secondary progressive multiple sclerosis. J. Neuroimmunol. 2000 Jul 24;107(2):130-9.
17. Kerlero de Rosbo N, Milo R, Lees MB, Burger D, Bernard CC, Ben-Nun A. Reactivity to myelin antigens in multiple sclerosis. Peripheral blood lymphocytes respond predominantly to myelin oligodendrocyte glycoprotein. J. Clin. Invest. 1993 Dec;92(6):2602-8.
18. Kerlero de Rosbo N, Hoffman M, Mendel I, Yust I, Kaye J, Bakimer R, Flechter S, Abramsky O, Milo R, Karni A, Ben-Nun A. Predominance of the autoimmune response to myelin oligodendrocyte glycoprotein (MOG) in multiple sclerosis: reactivity to the extracellular domain of MOG is directed against three main regions. Eur. J. Immunol. 1997 Nov; 27(11):3059-69.
19. Zhang J and Raus J, T-Cell vaccination trial in multiple sclerosis. “*T-Cell Vaccination and Autoimmune Disease*.” R.G. Landes Company, Austin, Texas,USA,1995, Chapter8, 135-71.
20. Zhang, J., Medaer, R., Hashim, G., et. al., Myelin basic protein specific T lymphocytes: precursor frequency, fine specificity and cytotoxicity. Ann. Neurol.1992, 32, 330-8.

**APPENDIX I**

**Relapsing-Remitting MS:**

Relapsing-remitting MS is characterized by clearly defined acute attacks with full recovery, or with sequlae and residual deficit upon recovery. (Fred D. Lublin, MD and Stephen C. Reingold, Ph.D. Defining the clinical course of multiple sclerosis. Neurology 1996; 46:907-911).

**Relapse Documentation Prior to Study Entry**

Every attempt will be made to obtain documentation of relapses which occurred one year prior to study entry. If no written documentation is available, the Investigator will contact the patient’s physician by phone in order to obtain all information of the patient’s relapses. This should be documented as a phone-call report.

**Definition of Relapse During the Study**

The following: “ relapse,” exacerbation,” “attack,” and “ bout” are equivalent terms. The term “**relapse**” will be used through this Protocol. A relapse is defined as the appearance of one or more new neurological abnormalities or the reappearance of on or more previously observed neurological abnormalities. This change in clinical state must last at least 48 hours and be immediately preceded by a relatively stable or improving neurological state in the 30 days before deterioration. An event is counted as a relapse only when the patient’s symptoms are accompanied by observed objective changes on the neurological examination consistent with an increase of at least 0.5 on the EDSS or one grade in the score for two or more of the seven functional systems (FS) of Kurtzke or two grades in the score for one of the FS and compared to the previous evaluation. The patient must not be undergoing an acute metabolic change such as fever or other medical abnormality and a change in bowel, bladder, or in cognitive function must not be entirely responsible for the changes in EDSS or FS scores.

**APPENDIX II**

**Instructions for Testing Lower Extremity Function:**

**Timed 10 Metter Walk**

MATERIAL NEEDED Stopwatch, 10 meter Walk Source Document Form,

Marked 10 meter distance in an unobstructed hallway,

Assistive Device (if needed)

TIME LIMIT PER TRIAL 3 minutes (180 seconds) per trial.

DISCONTINUE RULES 1) If the patient cannot complete a trial in 3 minutes

2) If the patient cannot complete Trial #2 of the Timed Walk after a 5 minute rest

ADMINISTRATION

*Trial 1*:

Make sure that the stopwatch is set to “0.” For the Timed 10 meter Walk, the subject should be directed to one end of a clearly marked 10 meter course (clearly defined on the floor or on the wall) and instructed to stand just behind the starting line. Point out where the 10 meter course ends, then instruct the patient as follows: “*I’d like you to walk 10 meter* *as quickly as possible, but safely. Do not slow down until after you’ve passed the finish line. Ready” Go.”*

Begin timing when the lead foot is lifted and crosses the starting line. The examiner should walk along with the patient as s/he completes the task. Stop timing when the lead foot crosses the finish line. The examiner should then record the subject’s walk time to within 0.1 second, round as needed. Round up to the next tenth if hundredths place is >0.05, round down if hundredth place is <0.05 (*e.g.* 32.45” would be rounded up to 32.5”, but 32.44” would be rounded down to 32.4”). Once the time is recorded, be sure to reset the stopwatch to “0.”

*Trial 2*:

After completing the first timed walk, position the patient just behind the line where s/he is now standing, repeat the same instructions, and have the patient complete the walk again.

*Assistive Devices*:

The goal is to use the same assistive device on each study visit. The patient will use any assistive device that s/he uses routinely, keeping in mind that the patient may deteriorate modestly during the trial. The study coordinator will enter this device on the Source Document Form. In general, patients should use their customary assistive devices(s), NOT the least assistive possible, to complete the test. For patients with significant gait impairment, the treating neurologist should have the patient use a rolling walker, even if this is not the patient’s customary device. Non-wheeled walkers may be used if this is the customary assistive device used by the patient.

COMPLETING THE SOURCE DOCUMENT FORM

*In the space provided on the source Document Form, record any circumstances that you believe may have affected the patient’s performance. These are factors that may have affected the trial, but were not severe enough to necessitate repetition of the trial. Examples include, but are not limited to the following:*

- *The patient had a cold or reports not feeling well;*
- *The patient tripped but did not fall.*

If a situation a situation arises that necessitates the repetition of a trial, check “Yes” for the question, “Did it take more than two attempts to get two successful trials?” and then specify the reason why a trial had to be repeated. Examples of reasons to repeat a trial include, but are not limited to, the following:

- The patient feel during the walk;
- The examiner forgot to start or to stop the stopwatch;
- The examiner forgot to reset the stopwatch between trials;
- The patient stopped to talk to someone while walking, or another person/thing somehow interfered with the walk.

**Record only the times for the**

**two successfully completed trials of the Timed 10 meter Walk.**

If the patient could not complete one or both of the trials for the Timed 10 meter Walk, record this in the appropriate section of the Source Document Form. If the patient’s disease has progressed and/or physical limitations prohibit him or her from completing the trial, you should check the box for “Unable to complete trial due to physical limitations.” Then record any specifics that you can observe (*i.e.*, patient is in a wheelchair now and is unable to walk, *etc.*). If the patient did not complete a trial for any other reason, check the box for “Other” and record the specific circumstances (*e.g.*, patient feel and was too fatigued to complete another trial, patient refused to complete trial).

**APPENDIX III**

**Ambulation Index**

| **Grade** | **Condition** |
| --- | --- |
| 0 | Asymptomatic; fully active. |
| 1 | Walks normally, but reports fatigue that interferes with athletic or other demanding activities. |
| 2 | Abnormal gait or episodic imbalance; gait disorder is noticed by family and friends; able to walk 25 feet (7.5 meters) in 10 seconds or less. |
| 3 | Walks independently; able to walk 25 feet in 20 seconds or less. |
| 4 | Requires unilateral support (cane or single crutch) to walk; walks 25 feet in 20 seconds or less. |
| 5 | Requires bilateral support (cane, crutches, or walker) and walks 25 feet in 20 seconds or less; or requires unilateral support but needs more than 20 seconds to walk 25 feet. |
| 6 | Requires bilateral support and more than 20 seconds to walk 25 feet; may occasionally use wheelchair. |
| 7 | Walking limited to several steps with bilateral support; unable to walk 25 feet; may use wheelchair for most activities. |
| 8 | Restricted to wheelchair; able to transfer self independently. |
| 9 | Restricted to wheelchair, unable to transfer self independently. |

The use of a wheelchair may be determined by lifestyle and motivation. It is expected that patients in Grade 7 will use a wheelchair more frequently than those in Grade 5 or Grade 6. Assignment of a grade in the range of 5 to 7, however, is determined by the patient’s ability to walk a given distance, and not by the extent to which the patient uses a wheelchair.

**APPENDIX IV**

**Magnetic Resoncance Imaging (MRI) Protocol**

T1-weighted (after Gadolinium injection) and dual-echo MRI scans of the brain will be performed. MRI will be acquired twice, *i.e.* at the baseline visit and at study termination. Therefore, the second MRI scan will be obtained 12 months after the baseline MRI scan.

Baseline MRI scans should always be obtained within 72 hours from the clinical baseline assessment.

The scanner is of 2.0 Tesla magnetic field strength.

Scanning Requirements

Given the importance of careful repositioning for serial MRI lesion counting and load assessments, great care should be taken when positioning the patients in the scanner and head holder. The following rules apply to all the centers:

- Check that the patient can successfully undergo MRI examination;

- Enter the patient data into the scanner console, using:

- - Patient’s initials;
  - Study number (protocol number, center number, patient number)
  - Date of birth
  - Sex
  - Weight
  - Scan number (use progressive numbers starting from 1 for the baseline scan, so for the project, scan #1 or scan #2).

- Explain the scanning procedure to the patient and position her/him in the scanner in the most comfortable position.

- **Insert an intravenous needle in the patient’s arm and connect it with a long-line catheter to a drip-infusion of saline; for contrast injection, the operator will be able to use the long-line without moving the patient table from the scanner.**
- Position the patient’s head, and align it in the machine using land-marking devices provided with most of the equipment;
- Position the patient’s eyebrows at the center of the coil, and make sure that the horizontal light beam runs over the eyebrows and as close as possible to the line marking the coil center;
- Position the patient’s nose along the Z axis of the scanner, *i.e.*, make sure that the vertical light beam runs over the nose;
- To avoid patient’s had movement, fix it with foam cushions and foal strips;
- Move the patient into the scanner;

Imaging:

The following order for sequences will be kept:

- T1-weighted axial scout;
- T1-weighted coronal scout;
- T1-weighted sagittal scout;
- Rapid T1-weighted SE or T2-weighted FSE/TSE axial scout (this is for repositioning check);
- T2-weighted FSE/TSE;
- T1-weighted SE post-gadolinium.

Sequences from 1-4 will be done to obtain reference images for patient repositioning.

Sequence parameters:

- TR: 100;
- TE: 10-20;
- Slice number: 3;
- Orientation: axial;
- Field of view (FOV): 210-230mm;
- Matrix: 128 x 256;
- Inter-slice gap: 5mm;
- Number of acquisitions: 1;
- Phase encoding: L > R.

Using sequence 1 as a reference, make a coronal scout, parallel to the brain transverse diameter and depicting the midline of the brain best (*i.e.* at the level of the brainstem); the acquisition parameters will be the following:

- TR: 100;
- TE: 10-20;
- Slice number: 1;
- Slice thickness: 5mm;
- Orientation: coronal;
- Field of view (FOV): 210-230mm;
- Matrix: 128-256;
- Number of acquisitions: 1;
- Phase encoding: L > R.

From the coronal scout, make a sagittal scout image, aligned with the *falx cerebri* and other midline structures, according to the following parameters:

- TR: 500-650;
- TE: 10-20;
- Slice number: 1;
- Slice thickness: 5mm;
- Orientation: sagittal;
- Field of view (FOV): 210-230mm;
- Matrix: 128 x 256;
- Number of acquisitions: 1-3;
- Phase encoding: A > P.

On the sagittal scout image previously obtained, position the axial image group, placing the center of the slice group at the inferior borders of the *corpus calosum genu* and *splenium*. The slice group will be positioned to include the whole brain from the vertex to the level of the *foramen magnum*. Save the localizer image with the slices shown on it, to achieve the same slice positioning on subsequent scans; then make a rapid series of either T-weighted or T2-weighted images (according to the site preference) in order to check the correct positioning of the slices.

For T1-weighted images, the following parameters should be used:

- TR: 600;
- TE: 10-20;
- Slice number: 22;
- Slice thickness: 3mm;
- Orientation: axial;
- Field of view (FOV): 250mm;
- Matrix: 140 x 256;
- Inter-slice gap: 3mm;
- Series: interleaved;
- Number of acquisitions: 1;
- Phase encoding: L > R.

For T2-weighted images, a fast spin echo (or turbo spin echo sequence should be used with the following parameters:

- TR: 1800-2000;
- TE: either single-echo or dual echo (according to the site preference):
  - TE first echo: 30-59;
  - TE second echo: 60-100;
- ETL: according to site preference;
- Slice number: 22;
- Slice thickness: 3mm;
- Orientation: axial;
- Field of view (FOV): 250mm;
- Matrix: approximately 14- x 256. The number of phase-encode lines will depend on the exact sequence used;
- Inter-slice gap: 3mm;
- Series: interleaved;
- Number of acquisitions: 1;
- Phase-encoding: L > R.

Please note that the hard copies of this first series of T1- or T2- axial images should be done only for the baseline scan and then used as a reference fore repositioning the second scan at the study termination visit.

Write the slice positioning parameters (shift and rotation angle) of this series and use them for the second scan this is not necessary if the scanner has a repeat or history function).

If possible, to speed up the examination, use a rectangular 3/4 FOV (*i.e.*, reduce the phase-encoding matrix by 25%) when doing all the subsequent series of axial images. When using 3/4 FOV, the FOV is 187.5 in L > R direction and 250mm in A > P direction; matrix is 256 (read) x 192 (phase).

The scanning procedure is resumed by doing two interleaved series of fast or turbo spin-echo dual echo sequence. Conventional spin-echo sequences are allowed in case fast imaging is not available.

Image parameters will be the as follows:

- TR: 2200-2800;
- TE: 15-50/80-120;
- ETL: 4-5;
- Slice number: 22;
- Slice thickness: 3mm;
- Orientation: axial;
- Field of view (FOV); 250mm;
- Matrix: 256 x 256 (192 x 256 if REC FOV);
- Inter-slice gap: 3mm;
- Series: interleaved;
- Number of acquisitions: 1
- Phase encoding: L > R;
- Re-saturation slab/flow compensations: Yes.

The axial pre-saturation slab (50-80mm) will be positioned inferior to the slice group to suppress flow-related artifacts.

The second series will have a slice position shifter 3mm caudally compared witht eh first one, so that when both series are combined, the whole brain is covered.

Some General Electric and Philips scanners allow all the two separate acquisitions (each of 22 slices) to be performed sequentially and automatically from a single prescription. If the scanner used allows the acquisitions to be split automatically in this way, then this should be done.

Perform a bolus injection of gadolinium-DPTA using the intravenous long-line and without moving the patient form the scanner. Use contrast at a standard dose of 0.1mMol/kg (*i.e.* 0.2ml/kg). During the post-injection delay interval the acquisition of additional sequences is allowed by negotiation with the MRI Steering Committee. After a post-injection delay of 5min, complete the canning with post-gadolimium T1-weighted SE images.

The following parameters should be used:

- TE: 15-50/80-120;
- ETL: 4-5;
- Slice number: 22;
- Slice thickness: 3mm;
- Orientation: axial;
- Field of vies (FOV): 250mm;
- Matrix: 256 x 256 (192 x 256 is REC FOV);
- Inter-slice gap: 3mm;
- Series: interleaved;
- Number of acquisitions: 1;
- Phase encoding: L > R;
- Re-saturation slab/flow compensation: Yes.

The second series will be positioned to fill the gaps of the fist one. Once the two acquisitions are completed, check the image consistency in terms of both correct repositioning (when compared with “fast” T1) and covering the whole brain as indicated above. In case of spoiled post-contract T1-weighted images due either to patient movement artifacts, or to a change in slice position of 6mm or more (*i.e.* > 2 slice thickness compared to baseline scan),k post-contrast T1-weighted sequence should be repeated within 20min after gadolimium DTPA injection.

When the scanning session of the MRI baseline scan is concluded, it is the responsibility of the treating neurologist to assess whether the abnormalities present on the MRI scans are compatible with MS (consultation with a local neuro-radiologist is advisable). If any other neurological condition is suspected on the basis of the MRI data, the patient will not be allowed to enter or to continue the study. Follow-up scans should be evaluated by the center radiologist and only non-MS changes may be discussed with the treating physician, if required.

**Steroid Treatment and MRI Scan Schedule**

It is recognized that steroid therapy strongly reduces the effect of gadolinium-enhancement and to a minor degree, the size of lesion of T2-weighted images. However, moderate and sever MS relapses may warrant treatment with I.V. methylpredisolone (IVMP), as described in an earlier section of this appendix. There is no best solution to this problem. In this study, patients treated with IVMP will be scanned at pre-planned times, rather than to arbitrarily adjust for recent steroid use.

On the MRI scans obtained at the termination of the study (all patients), the following analysis will be performed:

- Count of total contrast-enhancing T1-weighted lesions;
- Quantification of enhanced lesion volume;
- Count of new hyper-intense T2 lesions;
- Quantification of hyper-intense T2 volume;
- Quantification of hypo-intense T1 lesion volume;
- Quantification of brain volume and atrophy.

Three experienced neuro-radiologists will be in charge of lesion identification and counting in the study. For each scan, two of them (the same for the same patients throughout the entire study duration) will assess abnormalities by consensual agreement. In case of persistent disagreement (between the two neuro-radiologists), a fourth senior observer will make the final decision. A single enhancing lesion is defined as an area of enhancement seen on a given 3mm axial image, which is referable neither to normally enhanced structures, nor to contrast migration within vessels. T2-weighted FSE/TSE images will be used as reference for analysis. A single T2 lesion is defined as an area of increased signal on a given 3mm axial image, which should be seen on both T2- and proton density weighted images and which is not referable to normally hyper-intense structures. New T2 lesions have to appear in areas where on the previous scan no abnormality was detected. A single hypo-intense T1 lesion is defined as an area on a given 3mm axial slice with a signal intensity between those of gray matter and CSF. New T1 hypo-intense lesions have to appear in areas where on the previous scan no discrete hypo-intense abnormality was detected. Lesions that are contiguous in adjacent 3mm axial slices will be counted only once.

Lesion load and brain volume measurements will be done by trained technicians following reference hard-copies where the lesions were marked by a neuro-radiologist. The same technician will measure abnormalities and brain volumes on the scans from the same patients throughout the entire study duration. The image analysis will be performed using a semi-automated segmentation MRI Scan Quality Control.
